# The role of the west-dipping collision boundary fault in the Taiwan 2022 Chihshang earthquake sequence

**DOI:** 10.1038/s41598-023-30361-0

**Published:** 2023-03-02

**Authors:** Shiann-Jong Lee, Ting-Yu Liu, Tzu-Chi Lin

**Affiliations:** 1 Institute of Earth Sciences, Academia Sinica, Taipei, 115 Taiwan; 2 Department of Geosciences, National Taiwan University, Taipei, 106 Taiwan

**Keywords:** Seismology, Solid Earth sciences, Tectonics

## Abstract

On 17–18 September 2022, an earthquake sequence with a moment magnitude of 6.6 foreshock and a 7.0 mainshock occurred in southeast Taiwan along the Longitudinal Valley. Several surface breaks and collapsed buildings were observed after the event and one person died. The focal mechanisms of the foreshock and mainshock both had a west-dipping fault plane, which is different from the known active east-dipping boundary fault between the Eurasian Plate and the Philippine Sea Plate. Joint source inversions were performed to better understand the rupture mechanism of this earthquake sequence. The results show that the ruptures mainly occurred on a west-dipping fault. In the mainshock, the slip originated from the hypocenter and propagated toward the north with a rupture velocity of approximately 2.5 km/s. The east-dipping Longitudinal Valley Fault also ruptured, which could be passive and dynamically triggered by the significant rupture on the west-dipping fault. Most importantly, this source rupture model together with the occurrence of large local earthquakes over the past decade strongly supports the existence of the Central Range Fault, which is a west-dipping boundary fault that lies along the north to south ends of the Longitudinal Valley suture.

## Introduction

**Figure 1:**
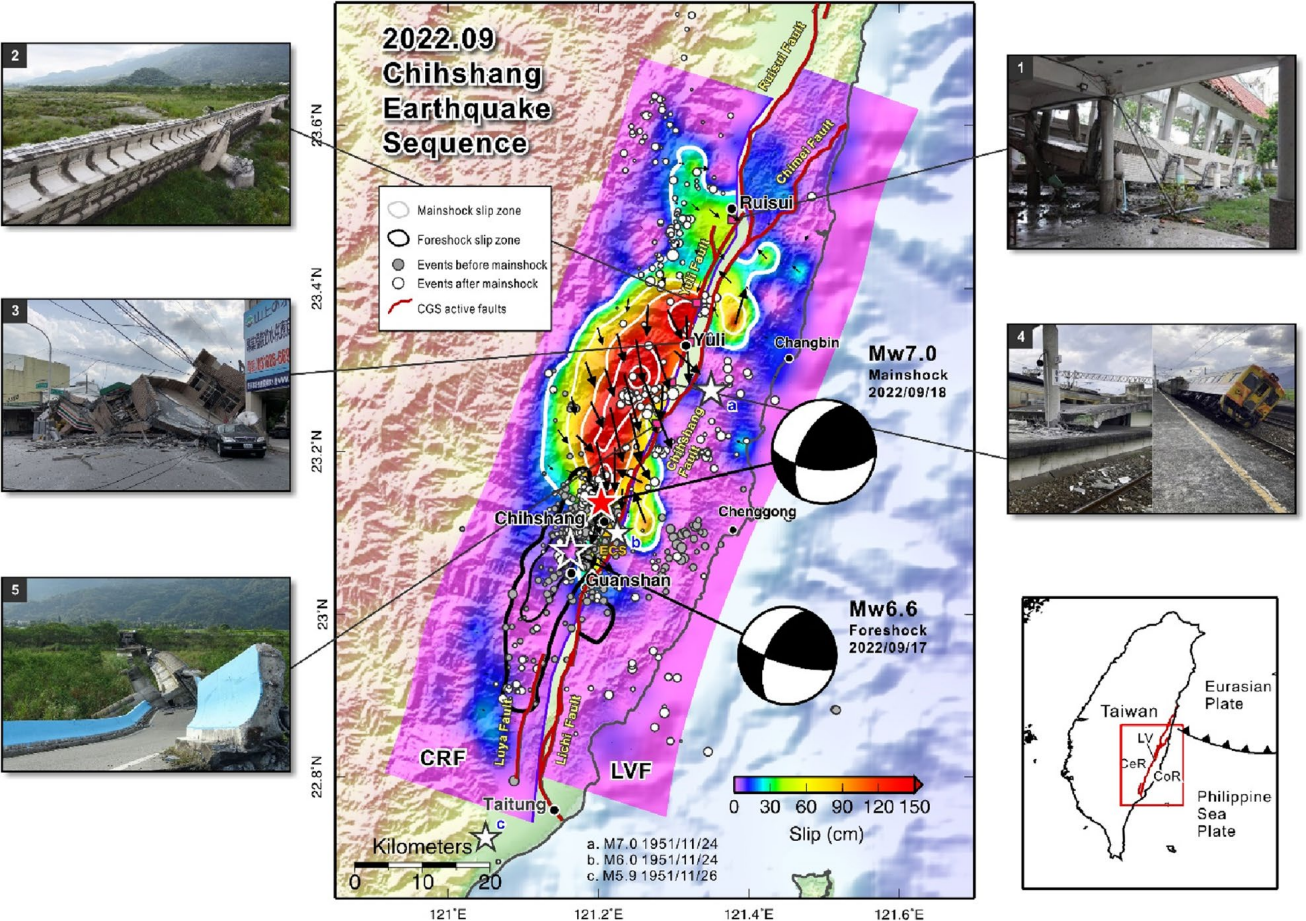
Map view of the slip distribution of the 2022 Chihshang earthquake sequence. Red solid star and white open star are the epicenter of 0918 mainshock and 0917 foreshock reported by CWB, respectively. White solid stars are the three events that occurred in the southern LV in the 1951 earthquake sequence (see Supplementary Table S1). The fault plane on the right is the east-dipping Longitudinal Valley Fault (LVF), and on the left is the west-dipping Central Range Fault (CRF). Active faults published by CGS^36^ are shown in red lines. White contours show the slip distribution of the mainshock, and the black contours indicate the slip area of the foreshock. Arrows are the slip vectors on the fault plane of the mainshock. Beachballs are the focal mechanisms determined by RMT^8^. The events before the 0918 mainshock (from 2022/09/17 to 2022/09/18 06:35 that were detected by CWB^37^) are shown in solid gray circles, and the events after the mainshock (from 2022/09/18 06:44 to 2022/09/30 19:51) are presented with white circles. Yellow triangle shows the station ECS that recorded an intensity of 6 + . The lower right illustrates the study area, with CeR representing the Central Range, CoR the Coastal Range, and LV the Longitudinal Valley which is the boundary between Eurasian Plate and Philippine Sea Plate. Photos 1–5 show the damages along LV caused by this earthquake sequence: (**1**) Chunrih elementary school (photo from Liberty Times, Taiwan), (**2**) Gaoliao bridge (photo from Chinese Television System, Taiwan), (**3**) 7-Eleven Yuli store (photo from United Daily News, Taiwan), (**4**) Dongli railway station (photo from ETtoday News, Taiwan), and (**5**) Luntian bridge (photo from Next Apple News, Taiwan). The map was generated by the GMT v.4.3.1 (https://www.generic-mapping-tools.org/).

The Chihshang earthquake, a large earthquake with a local magnitude of 6.8 (Mw 7.0), struck Taitung in eastern Taiwan on September 18, 2022 (Fig. 1, see also Table 1). One day before this event a strong foreshock with a local magnitude of 6.6 occurred, referred to as the Guanshan earthquake. These two events and the subsequent aftershocks are referred to as the 2022 Chihshang earthquake sequence. Both the mainshock and foreshock resulted in a maximum seismic intensity of 6 + (440–800 cm/sec^2^), which was recorded by station ECS in Chihshang Township. This is the first time that an earthquake with an intensity of 6 + has been observed since the Central Weather Bureau (CWB) in Taiwan adopted the modified intensity scale^1^. Unfortunately, one person died and many injuries and incidents of building damage were reported by Central Geological Survey^2^.

**Table 1:** List of large earthquakes that occurred along the LV suture in eastern Taiwan from 2013 to 2022.

LV	Date (yyyy/mm/dd)	Time (hh:mi:ss)	Longitude (°E)	Latitude (°N)	Depth (km)	M_L_	Mw	Strike (°)	Dip (°)	Rake (°)
North South	2018/02/06	15:50:41	121.73	24.10	6.31	6.26	6.4	215.7	56.4	25.6
	2019/04/18	05:01:07	121.54	24.06	18.8	6.32	6.2	204.0	63.0	66.0
	2021/04/18	14:14:37	121.48	23.86	13.9	6.26	6.2	199.9	52.6	53.7
	2014/05/21	00:21:13	121.43	23.74	16.5	5.99	5.9	208.1	60.2	58.5
	2013/10/31	12:02:09	121.35	23.57	15.0	6.42	6.5	209.2	59.4	51.4
	2022/09/18	06:44:15	121.19	23.14	7.81	6.83	7.0	205.0	61.3	46.6
	2022/09/17	13:41:19	121.16	23.08	8.61	6.60	6.6	199.5	72.0	29.3

The event time, location, and local magnitude are provided by the CWB earthquake catalog^37^. The moment magnitude and focal mechanism are taken from the RMT CMT report^8^.

Several aftershocks occurred from the foreshock event time to 30 September 2022. In total, 611 events with a minimum local magnitude of 0.98 were detected by CWB. The foreshock, mainshock, and most aftershocks were located on the western side of the Longitudinal Valley (LV) suture at depths between 5 and 20 km. However, some events occurred on the eastern flank of the LV at depths between 7 and 25 km. The distribution of this earthquake sequence is consistent with the strike of the LV, both being in the NNE to SSW direction (Fig. 1). It should also be noted that the events before the mainshock mostly occurred in the southern region of the mainshock epicenter. By contrast, the majority of events after the mainshock happened in the northern region from the mainshock epicenter.

The 2022 Chihshang earthquake sequence occurred in the LV, which is a suture zone between the Philippine Sea Plate and Eurasian Plate^3–5^. The centroid moment tensor of the mainshock and foreshock^4^ both show a reverse fault mechanism with left-lateral movement on a west-dipping fault plane (Fig. 1); however, the known active Longitudinal Valley Fault (LVF) system near this earthquake sequence, including the Chihshang and Guanshan Faults, dips toward the east.

The LV suture is one of Taiwan’s most active areas with a high level of seismicity, but it is observed that no large earthquake (Mw > 6.0) has occurred in the southern part of the LV within the last decade. However, in 1951, a series of earthquakes with three larger than magnitude 7.0 happened between Hualien and Taitung along the LV. These events are known as the 1951 Longitudinal Valley earthquake sequence^6,7^ (the list of 7 main events in 1951 can be found in Supplementary Table S1). The location of the 2022 Chihshang earthquake was near one of these 1951 large events (Fig. 1). After the Chihshang mainshock, people worried about the possibility of other big events might happen in the middle or northern LV like the 1951 Longitudinal Valley earthquake sequence.

To better understand the mechanism of the 2022 Chihshang earthquake sequence, a joint source inversion was performed using local seismograms, Global Navigation Satellite System (GNSS) coseismic displacement, and teleseismic body waves to analyze the rupture processes. The results further identify which structures were involved in this earthquake sequence and the relationship between the foreshock and the mainshock. Two candidate fault planes are considered (Fig. 1). One is the west-dipping Central Range Fault (CRF) plane, which is based on the focal mechanism derived from the Real-time Moment Tensor (RMT)^8^, the aftershock distribution, field surface breaks^2^, and damaged buildings (Fig. 1). Due to the presence of several surface breaks observed along the Chihshang Fault, an east-dipping LVF fault plane^9^ compiled by the Taiwan Earthquake Model (TEM) was selected as the second candidate in the inversion to evaluate its possible contribution to this event.

## Results

### Spatial distribution of the slip

**Figure 2:**
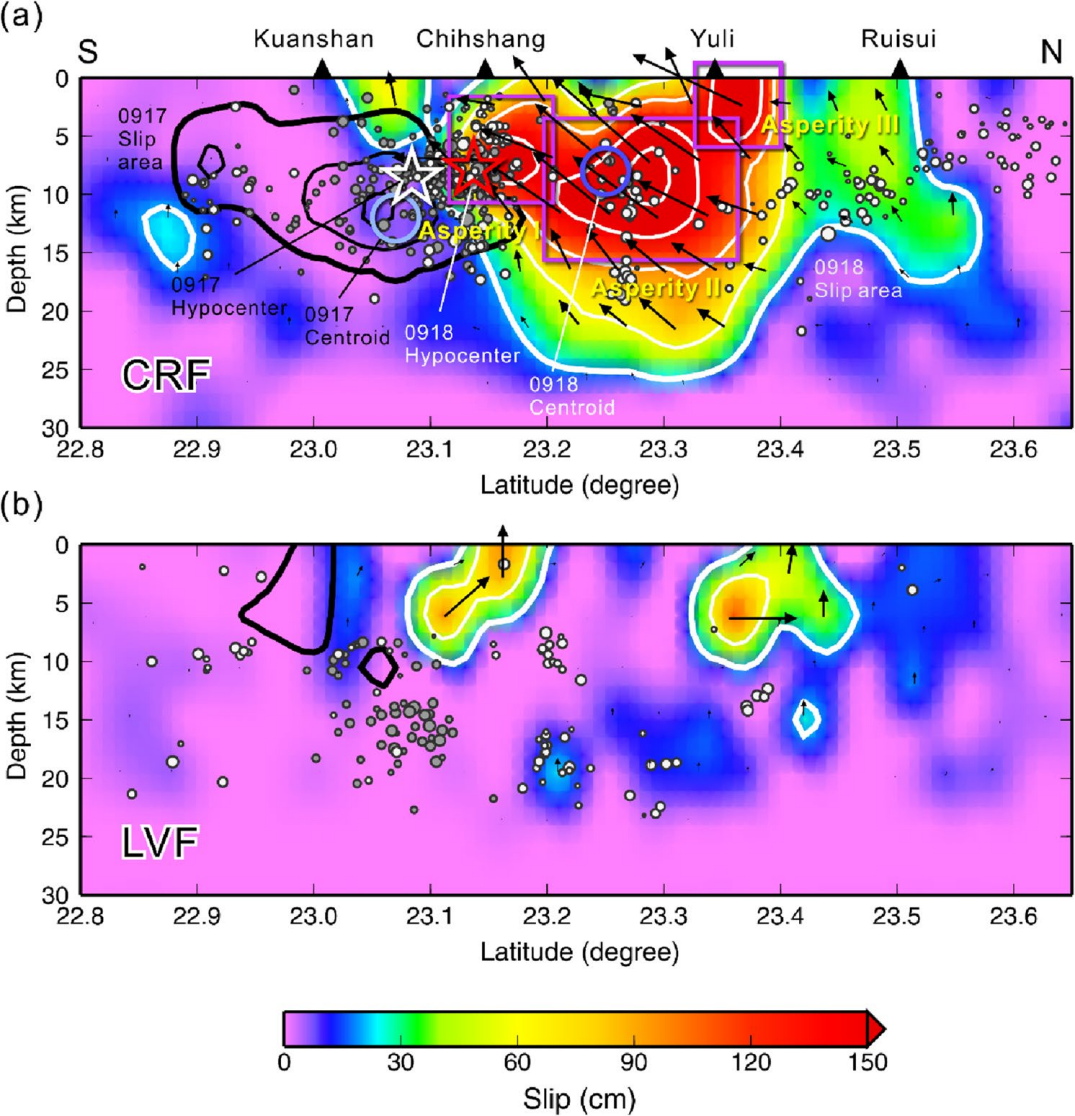
Distribution of slip on the: (**a**) west-dipping Central Range Fault, and (**b**) east-dipping Longitudinal Valley Fault. Red and white open stars are the hypocenters of the mainshock and foreshock, respectively. Dark and light blue open circles are the centroid locations of the mainshock and foreshock determined by the Real-time Moment Tensor monitoring system^4^ (https://rmt.earth.sinica.edu.tw). White contours are the slip distribution of the mainshock, and the black contours show the slip of the foreshock. The minimum threshold for contours is 20 cm, the second and third minimums are 60 and 100 cm respectively, and increments every 50 cm thereafter. Purple rectangulars indicate the three asperities on the fault plane. The events before the 0918 mainshock (from 2022/09/17 to 2022/09/18 06:35) are shown in solid gray circles, and the events after the mainshock (from 2022/09/18 06:44 to 2022/09/30 19:51) are presented with white circles. This aftershocks information is provided by CWB earthquake catalog^37^.

The slip distributions of the 0917 foreshock and 0918 mainshock are shown in Figs. 1 and 2, and detailed data fittings are provided in Supplementary Figs. S1–S3. Both main slips occurred on the west-dipping CRF. The foreshock slip area is compact and ruptured beneath the hypocenter, slightly propagating toward the south of approximately 20 km from the epicenter, but the mainshock ruptured over a large area on the northern fault plane. Three asperities were found in the mainshock slip zone (Fig. 2). Asperity I is located near the epicenter at a depth between 5 and 10 km. Asperity II lies much deeper in a very large area, with a size of about 10 × 15 km^2^ between 5 and 15 km depths, and extends northward. Also, Asperity II is the largest asperity of the mainshock, with a maximum slip of approximately 225 cm. Asperity III is a compact slip patch with a size of approximately 5 km × 5 km located in the north, and its slip is very close to the ground surface. A large area with moderate slip extends to deeper crust approximately 15–25 km below the three asperities. Some weaker slips (less than 50 cm) were also found in the northernmost fault plane (between 23.4°N and 23.6°N). Overall, the entire slip zone is approximately 50 km long along strike and 30 km wide in the downdip direction, with a maximum slip of 225 cm and an average slip of 82 cm. The stress drop determined by assuming the rupturing fault plane as a rectangular area for slip over 10% of the maximum slip is 1.05 MPa.

The slip on the CRF of the 0917 foreshock is more straightforward, with only one asperity beneath the hypocenter and some slip extending to the south close to the ground surface. In particular, both the foreshock and mainshock showed significant slips on the east-dipping LVF. These were located near Asperity I and Asperity III of the mainshock, and in the southern section of the foreshock. These slips are very shallow and close to the ground surface along the known LVF trace, i.e. the Chihshang Fault and Lichi Fault (Fig. 1).

Combining the slips of the foreshock and mainshock, the rupture zone covers a broad area nearly 60 km long along the LV suture that extends from the southern part of the LV close to Taitung to the middle of the LV close to Ruisui. In the map view (Fig. 1), the 2022 earthquake sequence can be seen to trend from southwest to northeast. The aftershocks mostly occurred out of the large asperities, with some located on the northernmost fault plane. A large number of the events occurred before the 0918 mainshock were located between the foreshock and mainshock slip zones. Some events were found on the eastern flank of the LV suture, which might have been related to activity on the LVF.

### Time history of the rupture

**Figure 3:**
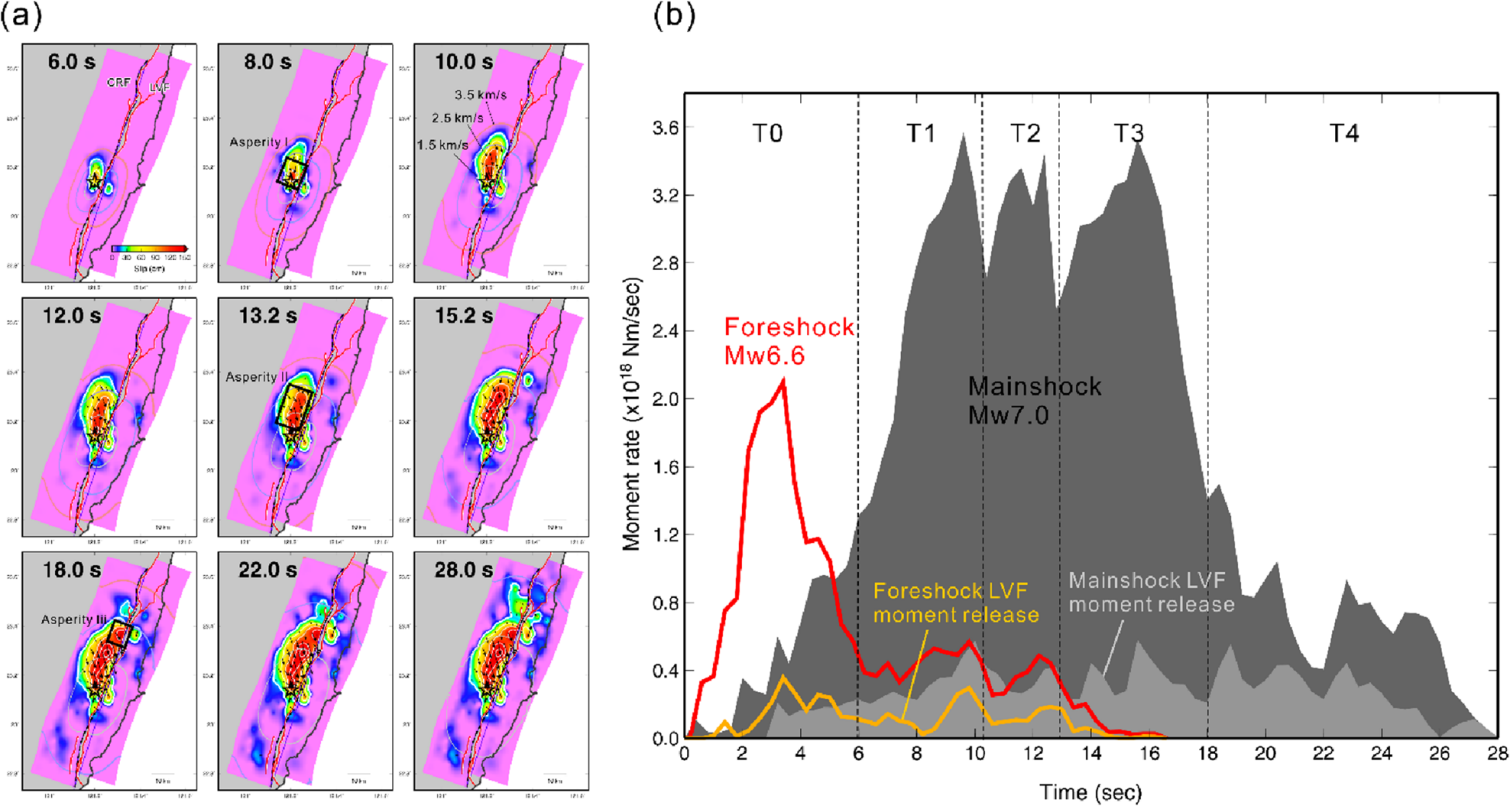
Rupture time history of the mainshock. (**a**) Rupture snapshots of the 0918 mainshock. Three reference rupture fronts with constant rupture speed Vr = 3.5, 2.5, and 1.5 km/s are shown with pink, blue and gray contours, respectively. Black rectangular indicates the three asperities. (**b**) The moment rate functions. The dark gray area shows the total moment release time history of the mainshock, and the light gray area indicates the moment released from the LVF. The moment rate function of the foreshock is shown with the red line, and the yellow line indicates the moment released from LVF. Five time intervals of the seismic moment releases are marked with T0, T1, T2, T3, and T4.

The rupture process of the mainshock was complex (Fig. 3a) with at least three energy bursts, as shown in the moment rate function (Fig. 3b). The slip between 0 and 6 s is small less than 80 cm, representing the weak initial rupture around the hypocenter. The first energy burst occurred between 6 and 10 s, and then the rupture started to propagate northward forming the first asperity near the hypocenter. The second energy burst lasted from 10 to 13 s. The rupture in this time period propagated to deeper crust between depths of 5 km and 18 km and quickly extended to the north. The third energy burst occurred between 13 and 18 s, and the rupture continued to develop in deep crust for more than 4 s (from 14 to 17 s) to form Asperity II. Almost at the same time (from 16 to 18 s), the rupture extended further toward the shallow northern fault plane that formed a compact slip area (Asperity III) close to the Yuli Fault. After 18 s, the slip grew mainly at the northern tip of the CRF fault plane but significant slips also occurred on the LVF before the completion of the rupture. It is noted that seismic energy was released continuously from the LVF between 3 and 28 s and gradually dominated the source time function before the rupture stopped. The rupture process (Supplementary Fig. S4) and moment rate function (Fig. 3b) of the foreshock are simpler compared with the mainshock; however, it also shows continuous seismic energy being released from the LVF. The duration of the moment release of the mainshock was approximately 28 s, and its total moment was 4.36 × 10^19^ N·m, equivalent to Mw 7.03. The seismic moment contributes from the LVF is 7.26 × 10^18^ N·m, roughly 17% of the total moment. For the foreshock, the total moment is 9.85 × 10^18^ N·m, which is equivalent to Mw 6.60.

To assess the fault rupture velocity, we considered the three reference rupture fronts with constant rupture speed Vr = 3.5, 2.5, and 1.5 km/s shown in Fig. 3. The deep rupture on Asperity II happened very quickly with slip between the 3.5 km/s and 2.5 km/s reference fronts. This indicates that the rupture on the deeper fault plane developed at speeds close to 3.0 km/s where the velocity of shear wave (Vs) in the middle crust is approximately 3.5–4.0 km/s. Therefore, the rupture on Asperity II is a normal subshear speed. On the other hand, the ruptures that developed in the shallow fault plane (Asperity III) were slower, rupturing after the 2.5 km/s rupture front. On the LVF, the rupture speed was also relatively slow, which originating quickly after the 3.5 km/s rupture front and then mainly propagating toward the ground surface with a speed of roughly 1.5–2.5 km/s.

### Individual inversion results

Inversions considering only teleseismic body waves, local strong motion, and GNSS coseismic displacement were performed to understand their individual contribution to the mainshock source model. The results show that the detailed slip patterns derived from the three data sets are different, but their main characteristics are consistent (Supplementary Fig. S5). The three source models all show that (1) the main slip zone occurred on the west-dipping CRF, (2) the rupture propagated to the north, and all asperities were found on the northern CRF fault plane, and (3) the shallow part of the LVF also experienced slip during the mainshock. Based on the fact that the same slip characteristics were found in the inversion results of all three data types, even though their station distribution and azimuth coverage are different, it is confirmed that these phenomena did indeed occur during the source rupture. Note also that the three data sets provide essentially different properties for the source inversion. The GNSS coseismic deformation data constrain the outline of slip pattern, and the local and teleseismic waveforms provide constraints on the history of rupture process. With these different characteristics derived from the three data sets, the comprehensiveness of the source rupture model is improved, containing a wide range of frequency information from short-periods (based on waveform data) to very long-periods (based on coseismic displacement).

## Discussion

To evaluate the robustness of the source rupture model of the mainshock, we conducted a forward seismic waveform simulation using the inverted source model based on the spectral element method (SEM)^10,11^. An island-wide comparison between simulated and observed CWB24 waveforms is shown in Supplementary Fig. S6. The synthetic waveforms fit with the main features of the observed data for the frequency band between 0.05 and 0.5 Hz. The ground shaking was observed to be extreme in the source area, especially on the hanging wall along the CRF, e.g. stations F073 and G061. The station located in western Taiwan also recorded large amplitudes. However, some synthetic waveforms do not fit well with the observations, such as SHH, ICHU, and CHY. The poor fit could have been due to the presence of soft sediment that caused nonlinear effects^12–14^ and amplified the ground shaking further in the Western Plain. On the contrary, northern Taiwan suffered smaller ground shaking during the mainshock. The misfit of the overall 80 s vertical component displacement waveform is 0.378. The low misfit of the island-wide waveforms means that the source rupture model is precise to reproduce the displacement ground motion for periods of approximately 2 s.

**Figure 4:**
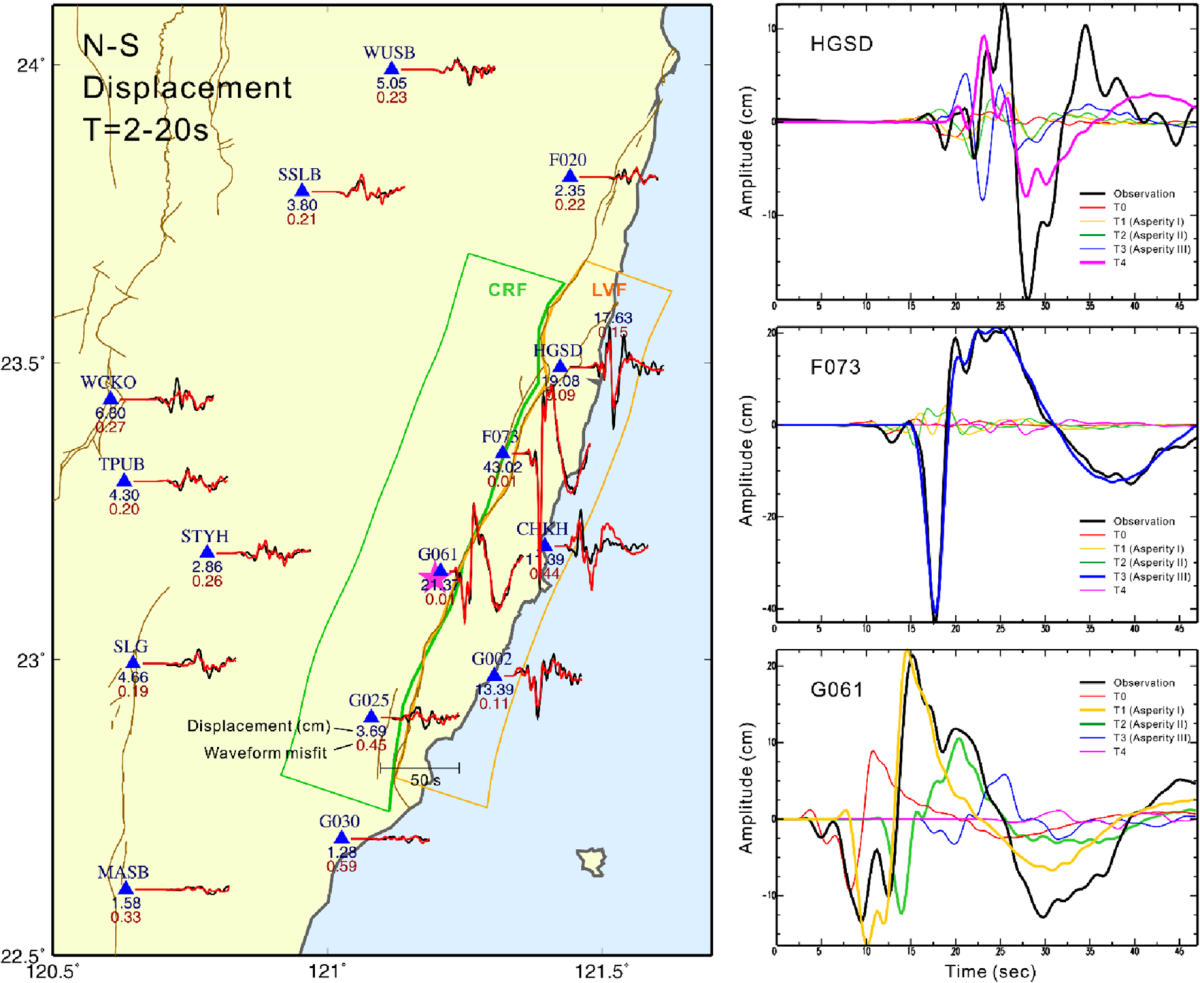
Comparison of the mainshock local observed and synthetic displacement waveforms in the north–south component. The black lines are observations and the red lines are synthetics. All the waveforms are bandpass filtered at 0.05 and 0.5 Hz. The blue and red numbers beneath each seismic station are the maximum amplitude (cm) and waveform misfit, respectively. The maximum amplitude is taken from the maximum absolute value of the whole waveform. The right panels show the contribution of five different time periods in the moment rate function (Fig. 3) to the near-field strong ground motion waveforms. The map was generated by the GMT v.4.3.1 (https://www.generic-mapping-tools.org/).

Furthermore, the synthetic waveforms were calculated individually according to different energy burst time periods to investigate the anomalously large, long-period ground shaking produced in the northern fault plane along the LV (Fig. 4). For station G061, which is closest to the epicenter, the long-period waveform resulted from the combination of Asperity I near the hypocenter and Asperity II where the slip occurred in the deeper crust. Station F073 recorded an anomalously large amplitude in the displacement waveform. This large, long-period phase was mainly contributed by Asperity III, which is a compact slip zone that coincides with the surface break observed along the Yuli Fault. Other asperities had less influence on the waveform recorded at this station. The HGSD station is located on the footwall of CRF near the northern tip of the Yuli Fault. The large phase recorded by this station is dominated by the slip that occurred on the LVF in which the rupture propagated slowly to the surface and produced a long-period signal in the seismic waveform. This long-period phase in the displacement has a pulse-like shape in the velocity waveform and is known to have been caused by the rupture directivity effect in which amplified long-period ground shakings are observed along the rupture direction^15–17^. From the comparisons of the stations along the LV, it is well established that the long period, large amplitude ground motion was caused by the various rupture areas rather than by a single large asperity. More precisely, the rupture directivity effect in most cases could have been related to the behavior of the nearby fault rupture.

The 2022 Chihshang earthquake sequence started from the foreshock of Mw 6.6, followed by 259 smaller events with a minimum local magnitude of 0.98, and then the Mw 7.0 mainshock occurred 17 h later as reported by CWB. The seismic moment released by the foreshock was approximately 25% of the mainshock. It is noted that the foreshocks were primarily located south of the mainshock epicenter, and the aftershocks occurred north of it (Fig. 1). The slip distribution shows a similar result, i.e. the slip zone of the Mw 6.6 foreshock extended to the south, whereas the mainshock primarily ruptured to the north of the epicenter (Fig. 1).

**Figure 5:**
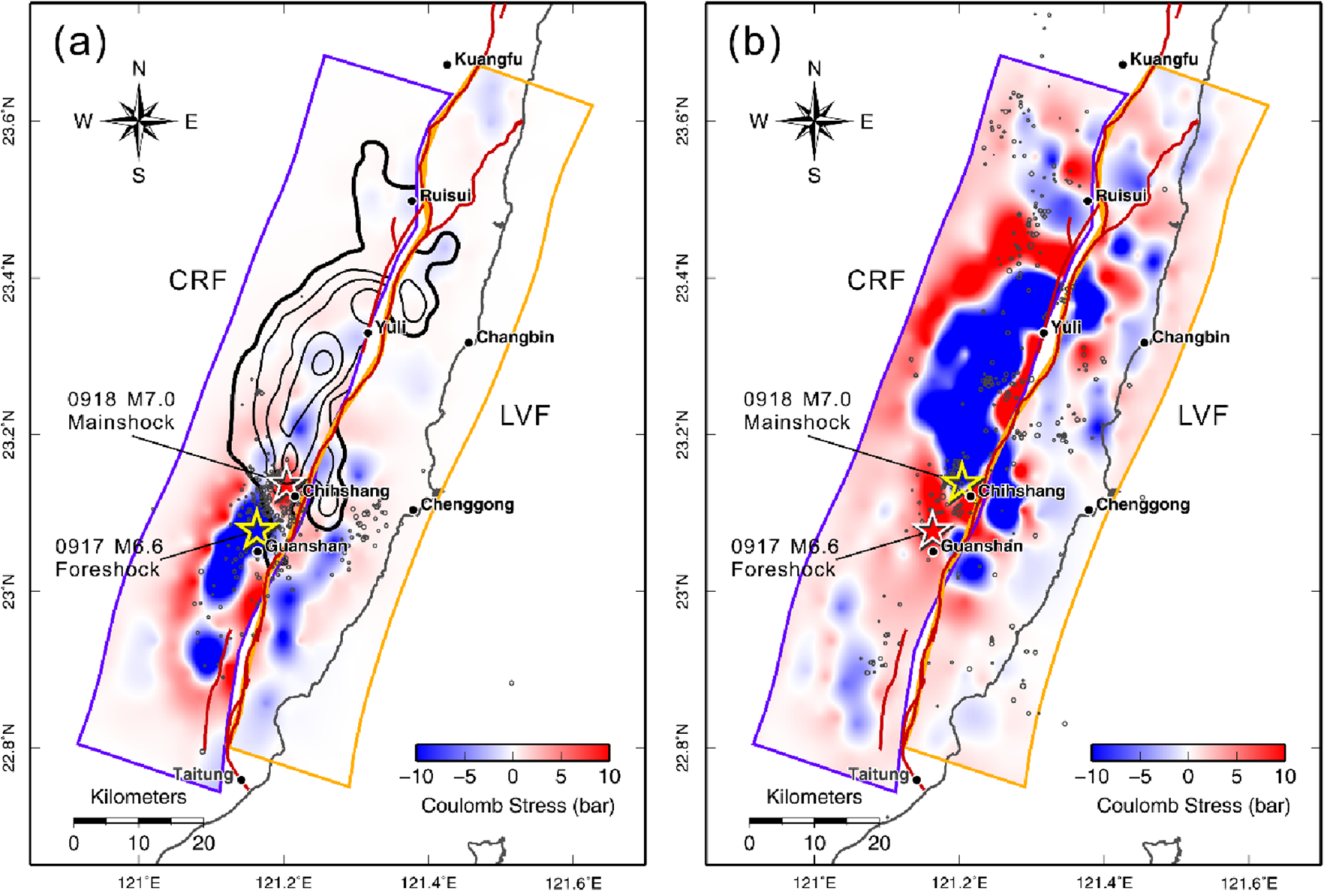
The Coulomb stress change caused by the 2022 Chihshang earthquake sequence. (**a**) The Coulomb stress change on the CRF and LVF that caused by the finite-fault source model of the 0917 foreshock. Black contours show the slip distribution of the mainshock. Events before 0918 mainshock are shown with open circles. The mainshock epicenter and most of the events before the mainshock are located in areas where △CFS increased significantly. (**b**) The Coulomb stress change on the CRF and LVF that caused by the 0918 mainshock. Open circles are events that occurred after the mainshock. The map was generated by the GMT v.4.3.1 (https://www.generic-mapping-tools.org/).

To further understand the relationship between the foreshock and mainshock in the 2022 Chihshang earthquake sequence, an analysis of the static Coulomb failure stress change (ΔCFS)^18^ on both the CRF and LVF fault planes using the foreshock source model was performed. The strike and dip angles of the receiver faults are varied according to the geometry of CRF and LVF (see Supplementary Table S2). The rake angle of the receiver fault for the CRF is 46.6° which was determined from the mainshock focal mechanism. The rake angle for the LVF was fixed at 45.0°. The results show that the mainshock hypocenter and the nearby Asperity I are located in the area where ΔCFS increased significantly after the M6.6 foreshock (Fig. 5a). The shallow asperity near the Yuli Fault trace (Asperity III) and the start of deep asperity (Asperity II) are also located in areas where ΔCFS increased. This indicates that the slip pattern of the mainshock, especially the location of its nucleation and asperities, can be controlled within the area with increased ΔCFS caused by a strong foreshock. The events that occurred near the LVF fault plane before and after the mainshock were generally located in areas of increased ΔCFS as expected (Fig. 5b).

Significant slip occurred on both the CRF and LVF during the mainshock. The contribution to the seismic moment release of the LVF was 7.26 × 10^18^ N·m, equivalent to Mw 6.5 and approximately 17% of the total moment of the mainshock. The moment releases of the LVF lasted for a long time, from around 3 s to 28 s. The foreshock was similar in that the LVF released about 20% of the total seismic moment. From the rupture snapshot of the mainshock, it was found that the origin of the LVF slip started after the 3.5 km/s rupture front (Fig. 3, see also Supplementary Video S1), which is very close to the local S wave speed. These snapshot results imply that the rupture on the LVF could have been triggered dynamically when strong S waves propagated through the LVF. This is the reason why the rupture that originated on the LVF occurred quickly after the rupture front of 3.5 km/s passed. Once the rupture on the LVF was triggered, it propagated slowly at approximately 1.5–2.5 km/s to the weakly-coupled creeping region on the shallow fault plane where the fault tends to slip^19–22^.

The 2022 earthquakes are not the only case in which slip occurred simultaneously on both the CRF and LVF. For example, a fault-to-fault jumping rupture was found to have occurred during the 2018 Mw 6.4 Hualien earthquake^23^. The rupture originated from a NE-SW striking, west-dipping fault plane and then jumped to the shallower east-dipping Milun and Lingding Faults on the northernmost section of the LV. Thus, the 2018 event also ruptured on both the east-dipping sections of the LVF, i.e. the Lingding Fault, which is the northernmost segment of the LVF, and a west-dipping fault plane, which could possibly have been the northernmost tip of the CRF. As discussed previously, ruptures on the LVF could be triggered dynamically when strong S waves are excited by large asperities on the CRF and then propagate through the LVF. Thus, the interaction between these two faults during a large earthquake could be a common phenomenon that has probably not been noticed before because no significant surface ruptures had been observed. There could be more cases in which both the LVF and CRF ruptured simultaneously, especially during large earthquakes like the 1951 LV earthquake sequence, that can produce anomalously large ground shaking as well as strong directivity effects to trigger the rupture on nearby faults dynamically.

Over the past 10 years, an earthquake with a magnitude of 6.0 or greater has occurred in eastern Taiwan almost every 1 or 2 years. Starting from 2013, the Mw 6.4 Ruisui earthquake followed by the 2014 Mw 5.9 Feling earthquake occurred in the central area of the LV. After these two events, large earthquakes moved to the northern LV, including the 2018 Mw 6.6 Hualien earthquake, the 2019 Mw 6.2 Hualien earthquake, and the 2021 Mw 6.2 Shoufeng earthquake. These were followed by the 2022 Chihshang earthquake sequence in which the earthquake location had moved to the south LV. In all, seven big events have occurred along the LV (Table 1), and they share a common feature: their epicenter and main slip area are all located on a westward-dipping fault plane^23–25^. The CRF was first proposed by Shyu et al.^26^ based on geomorphic characteristics. However, the seismic activity along the CRF was less active before the 2013 Ruisui earthquake. After two earthquakes occurred in the southern section of the LV in 2022, the existence of a west-dipping fault plane from north to south along the LV has almost been confirmed.

**Figure 6:**
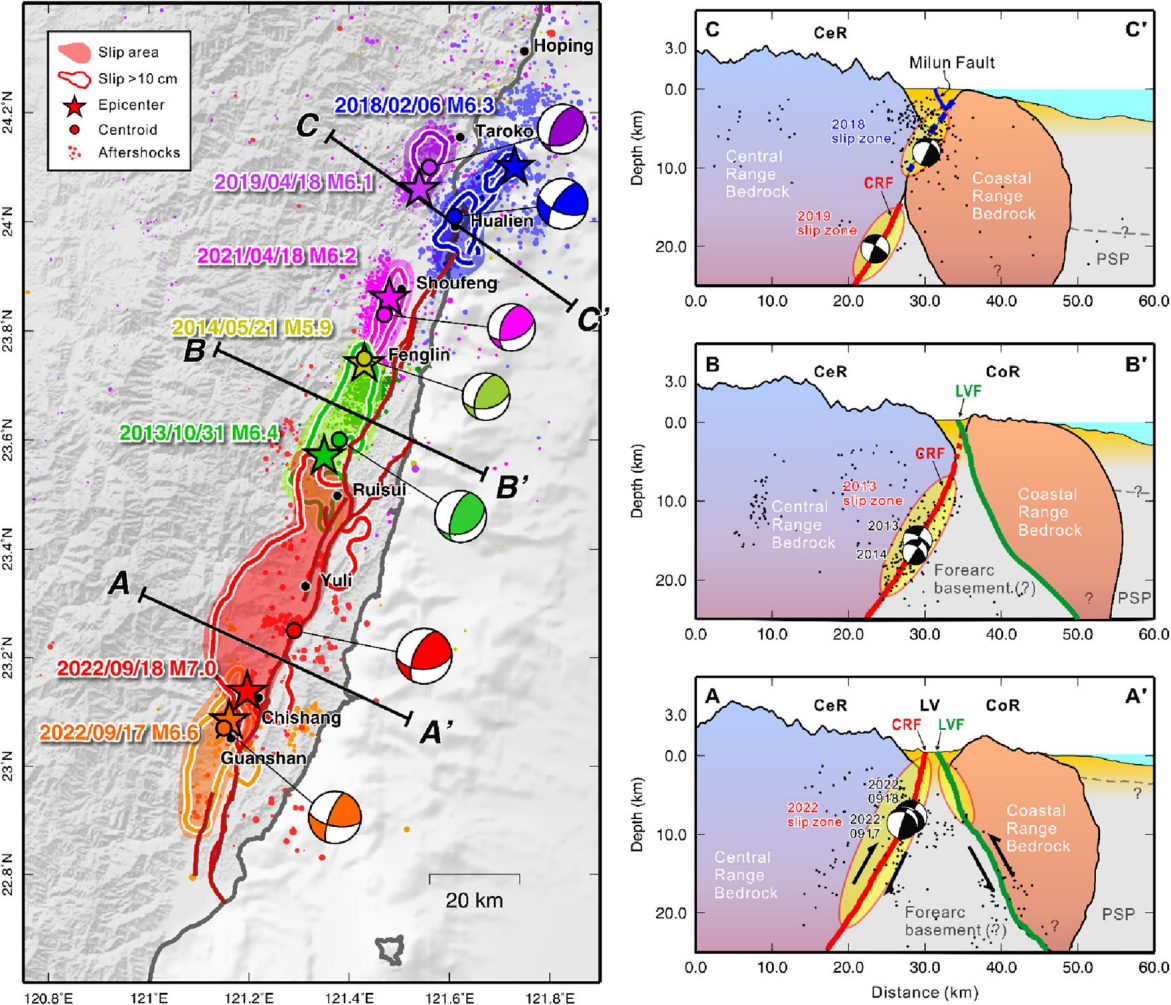
Big earthquakes occurred in eastern Taiwan along the LV from 2013 to 2022 (see also Table 1). The epicenters (stars) are given by the CWB earthquake report; the focal mechanisms and centroid locations of each event are taken from the RMT centroid reports^8^ (https://rmt.earth.sinica.edu.tw). The color contours indicate the slip areas of the large events based on the finite-fault source inversion studies^23–25^. The conceptual tectonic models for the CRF along the three profiles across the Longitudinal Valley suture from north to south are shown in the right panels. The red line indicates the west-dipping CRF, and the green line shows the east-dipping LVF. The dotted blue line indicates a shallow west-dipping fault plane where the rupture of the 2018 Hualien earthquake originated^23^. The map was generated by the GMT v.4.3.1 (https://www.generic-mapping-tools.org/).

Combining the geomorphic results^26^ and the seismic information, including the focal mechanisms and slip distribution of past large events^23–25^, along with their aftershocks, a conceptual tectonic model of the CRF across three profiles along the LV suture is provided in Fig. 6. The southernmost A-A’ profile lies across the rupture area of the 2022 Chihshang earthquake. Our inversion results show that slip extended from 25 km depth to near the ground surface that connects with the Yuli Fault, which might be related to the CRF. In the southern LV, many previous studies indicate that the CRF is presented as a high-angle west-dipping boundary between the Central Range (CeR) bedrock and the remnant of the forearc basement^27–29^, i.e. the lithosphere of the Philippine Sea Plate (PSP). Between the CRF and LVF (the Chihshan Fault), the remnant of the forearc basement is covered by alluvial sediments deposited as a shallow surface layer. Further east, the hanging wall of the LVF belongs to the Coastal Range (CoR) bedrock.

Profile B-B’ is located in the middle of the LV suture across the rupture zone of the 2013 Ruisui earthquake. This event had no clear evidence of surface break associated with CRF, and the slip mainly occurred below 5 km depth^24^. Accordingly, the top of the CRF in the middle LV could be deeper than 5 km below ground surface. The exact fault plane of the CRF at depths shallower than 5 km is unknown and may intersect the LVF. It is worth noting that the PSP starts to subduct toward the north underneath the Eurasian Plate (EP) at approximately 23.5°N^30^, which is close to the middle LV. This could result in the lithosphere of the PSP moving deeper, and thus the boundary between the CeR and PSP, i.e. the CRF, could migrate to the middle-to-deep crust. Profile C–C’ crosses the rupture areas of the 2018 and 2019 Hualien earthquakes in the northernmost section of the LV. In this area, the PSP is subducting at a depth of approximately 20 km. In the shallow part, the CoR bedrock is subducting northward beneath the Eurasian Plate together with the PSP. The origin of 2018 and 2019 events occurred on two different west-dipping faults, which are the boundaries between the CoR bedrock, CeR bedrock, and PSP^23,25^. These west-dipping boundaries could be the northern extension of the Central Range Fault.

It is noted that from 2013 to 2022, the slip areas of these large events almost filled the entire west-dipping fault plane. Thus, the two events in the 2022 Chihshang earthquake sequence can be said to be the last piece of the CRF puzzle. However, this boundary structure may extend further south along the coast of southeast Taiwan^26^ where no major earthquakes have been recorded since 1990. At the same time, the LVF on the east flank of the suture has experienced fewer large earthquakes since the 2003 Mw 6.4 Chengkung earthquake. Therefore, it is still necessary to be cautious when considering earthquake damage prevention and disaster reduction, especially in eastern Taiwan.

## Methods

### Data processing of the three data sets

In the joint source inversion analysis, three data sets are used, including local ground motion waveform records, GNSS coseismic displacements, and teleseismic P-body waves. The teleseismic P waveforms were obtained from the Global Seismic Network (GSN^31^), of which 31 records with high signal-to-noise ratio were selected for the mainshock, and 26 records were considered in the foreshock (see Supplementary Table S3). To avoid the complexity of the shallow crust, the epicentral distance of the used stations was constrained between 30° and 90° (Supplementary Fig. S1). The raw teleseismic velocity data (with a length of 5400 s, starting from 1800s before the event time) were first applied a four-pole, one-pass Butterworth band-pass filter with corners at 0.01 and 0.5 Hz. Then integrated once to obtain displacements and resampled to five points per second. Finally, a 45 s waveform time window, including 10 s before the P arrival, was cut from the processed waveform for the inversion in the mainshock. Considering a smaller magnitude and thus a shorter source duration, a 40 s waveform time window was used in the foreshock.

Local waveforms were compiled from the Taiwan Strong Motion Instrument Program (TSMIP), Central Weather Bureau 24-bit network (CWB24), and Broadband Array for Taiwan Seismology (BATS). There were 47 local waveforms in all three components used for the mainshock, as shown in Supplementary Fig. S2a. Due to larger background noise, there were 34 waveforms used for the foreshock (Supplementary Fig. S2b). The raw data were band-pass filtered from 0.05 to 0.5 Hz and then integrated to obtain displacements. A 50 s waveform at the origin time of the event was used in the inversion, with a sampling interval of 0.2 s.

GNSS data were compiled by the geodetic group in the Institute of Earth Sciences. Coseismic displacements were taken from 1 Hz high sampling rate continuous GNSS records. For the foreshock, the coseismic displacement was estimated from the position difference between the averages of 1 Hz data 30 s before and 30 to 60 s after the event time. The mainshock was estimated from the position difference between the averages of 1 Hz solutions one minute before and two minutes after the event time. There are 69 GNSS stations used in the inversion which are distributed on the whole Taiwan island, and each station has three components of coseismic displacements. (Supplementary Fig. S3). It is noted that the high sampling rate GNSS could provide continuous data with poor precision. However, the qualities of coseismic displacements of these two events are good. This might be because the ruptures were very close to the ground surface.

It is noted that we utilized different frequency bands for the three data sets according to their instrument responses and the characteristics of the data. Generally, the wider the frequency band used, the more the original data characteristics can be preserved. However, since the instrument response has a limited dynamic range and the data usually contains high-frequency background noise, different data must be filtered separately to extract corresponding source signals. Therefore, a bandpass filter between 2 and 100 s was considered for the broadband teleseismic data to contain long-period signals. For the local seismic waves, because the strong motion instrument cannot record precise long-period signals, a bandpass filter between 2 and 20 s was used. Finally, no filter was applied for the GNSS data to preserve information on permanent displacement.

### Joint source inversion

The observation equation of the joint source inversion is represented by *Ax* = *b* to determine the rupture process during the earthquake, in which three sets of observational data are arranged along vector *b*, the corresponding Green’s functions are set in matrix *A*, and the slip distribution is determined by the solution vector *x*. A misfit function defined by *(Ax − b)*^2^*/b*^2^ is applied to evaluate the quality of fitting between the synthetic and the observational data. Following the approach proposed by Hartzell and Heaton^32^, the inversions were considered with 24 multiple time windows each being 0.8 s long and with an overlap of 0.4 s, to improve the resolution of the rupture process. A detailed discussion about the spatial and temporal resolution of the inversion is provided in the Supplementary Methods.

To make the three data sets and inversion constraints contribute equally to the inversion, a normalized weight for each data set is given:

Normalized data weighting = 1/(Σ|Observed data value|/Number of data).

Following this rule, the normalized weightings for teleseismic, GNSS coseismic displacement, and local strong motion data are 0.8, 0.3, and 0.1 respectively. This nonlinear joint inversion problem was solved based on the parallelized non-negative least squares method developed by Lee et al.^24^ under a 24-node high-performance cluster.

The Green’s functions of the local waveforms and GNSS displacement in matrix *A* were calculated based on the SEM^10^ considering a three-dimensional tomographic structure^33^. For the teleseismic data, the Green’s functions were based on the Synthetics Engine (Syngine) for the 1D Earth reference model using the Preliminary Reference Earth Model (PREM)^34^ which is provided by IRIS Data Management Center^35^. For both local and teleseismic waveforms, the same filtered frequency band for the observed data was employed to the synthetics individually. The maximum rupture velocity (V_rmax_) of 2.8 km/s was set for the inversion, which is close to the local shear wave velocity near the source area of approximately 3.0–4.0 km/s. An analysis of misfits with varied V_rmax_ is provided in Supplementary Table S4. The result of V_rmax_ 2.8 km/s has the smallest total misfit 0.09. It also shows the smallest misfits in the three data sets, including teleseismic waveform, local ground motion, and GNSS coseismic displacement. According to this analysis, we choose the result with V_rmax_ = 2.8 km/s in this study.

## Supplementary Information

Supplementary Information.

Supplementary Video 1.

## Data Availability

The local seismic data, including TSMIP, CWB24, and BATS are available from the TEC data center (https://tecdc.earth.sinica.edu.tw/tecdc/). The teleseismic data can be downloaded from IRIS DMC (https://ds.iris.edu/ds/nodes/dmc/). The GNSS data are available from TEC Taiwan GPS web service at https://tec.earth.sinica.edu.tw/service.php?id=3#. The teleseismic body waveforms were taken from Incorporated Research Institutions for Seismology (IRIS) at https://ds.iris.edu/wilber3/find_event (last accessed Oct 2022).
