# Supplementary material for: The role of the west-dipping collision boundary fault in the Taiwan 2022 Chihshang earthquake sequence
Source: Sci Rep. 2023 Mar 2;13:3552. doi: 10.1038/s41598-023-30361-0 (PMC9981749; doi:10.1038/s41598-023-30361-0)
Supplement: Supplementary file 1 — Supplementary Information. [file 41598_2023_30361_MOESM1_ESM.docx]

Supplementary Information

**The role of the west-dipping collision boundary fault in the**

**Taiwan 2022 Chihshang earthquake sequence**

Shiann-Jong Lee^1*^, Ting-Yu Liu^1^, Tzu-Chi Lin^1,2^

^1^Institute of Earth Sciences, Academia Sinica, Taipei 115, Taiwan

^2^Department of Geosciences, National Taiwan University, Taipei 106, Taiwan

*Corresponding author

Shiann-Jong Lee ([sjlee@earth.sinica.edu.tw](mailto:sjlee@earth.sinica.edu.tw))

**This PDF file includes:**

Supplementary Figure S1 to S8

Supplementary Table S1 to S5

Caption for Supplementary Video S1

Supplementary Methods


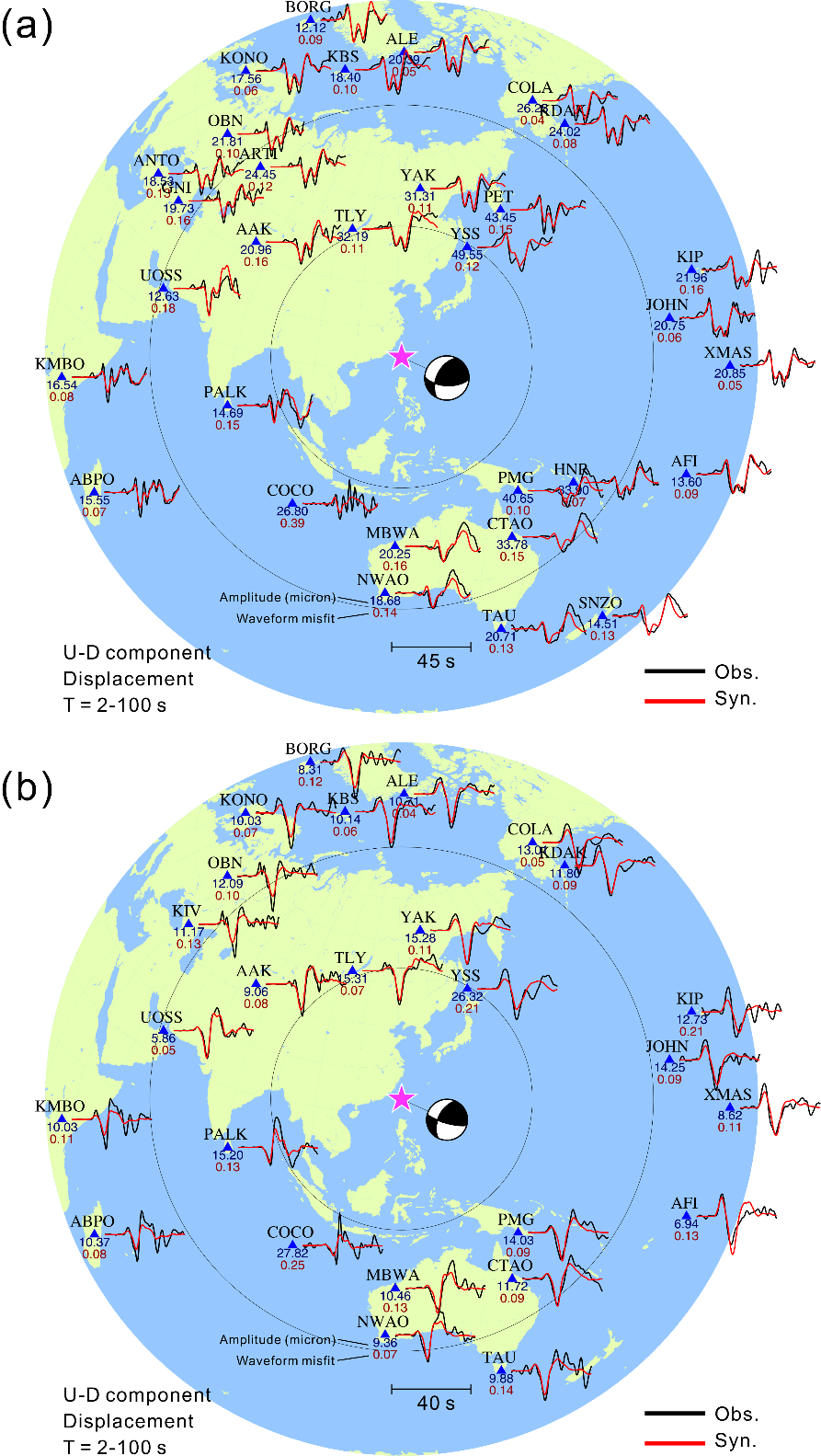


**Figure S1.** Comparison between the observed teleseismic P waves and synthetics: (a) 0918 mainshock, and (b) 0917 foreshock. Black lines are observations and red lines are synthetics. All the waveforms are displacement type starting from 10 seconds before P and ending at 30 seconds after the P arrival. A bandpass filtered between 0.01 and 0.5 Hz was employed. The blue and red numbers beneath each Global Seismic Network station are the maximum amplitude and waveform misfit, respectively. The beachballs show the focal mechanisms determined by the Real-time Moment Tensor (RMT)^8^. The map was generated by the GMT v.4.3.1 (https://www.generic-mapping-tools.org/).


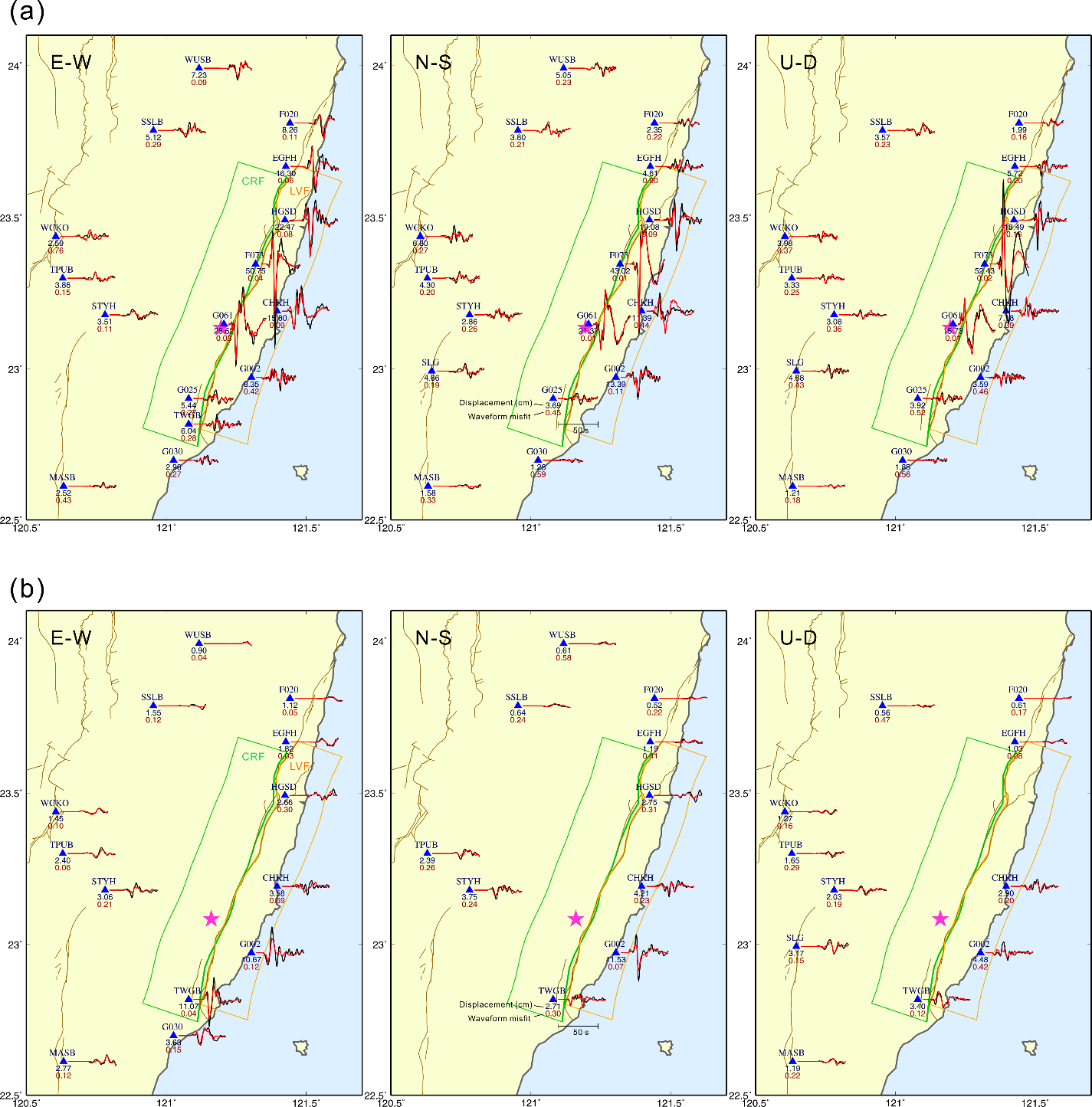


**Figure S2.** Comparison between the observed local ground motion waveforms and synthetics: (a) 0918 mainshock, and (b) 0917 foreshock. From left to right are E-W, N-S, and U-D components, respectively. Black lines are observations and red lines are synthetics. The blue and red numbers beneath each seismic station are the maximum amplitude and waveform misfit, respectively. All the waveforms are in displacement type, starting from event time and lasting for 50 seconds. A bandpass filtered between 0.05 and 0.5 Hz was applied. The map was generated by the GMT v.4.3.1 (https://www.generic-mapping-tools.org/).


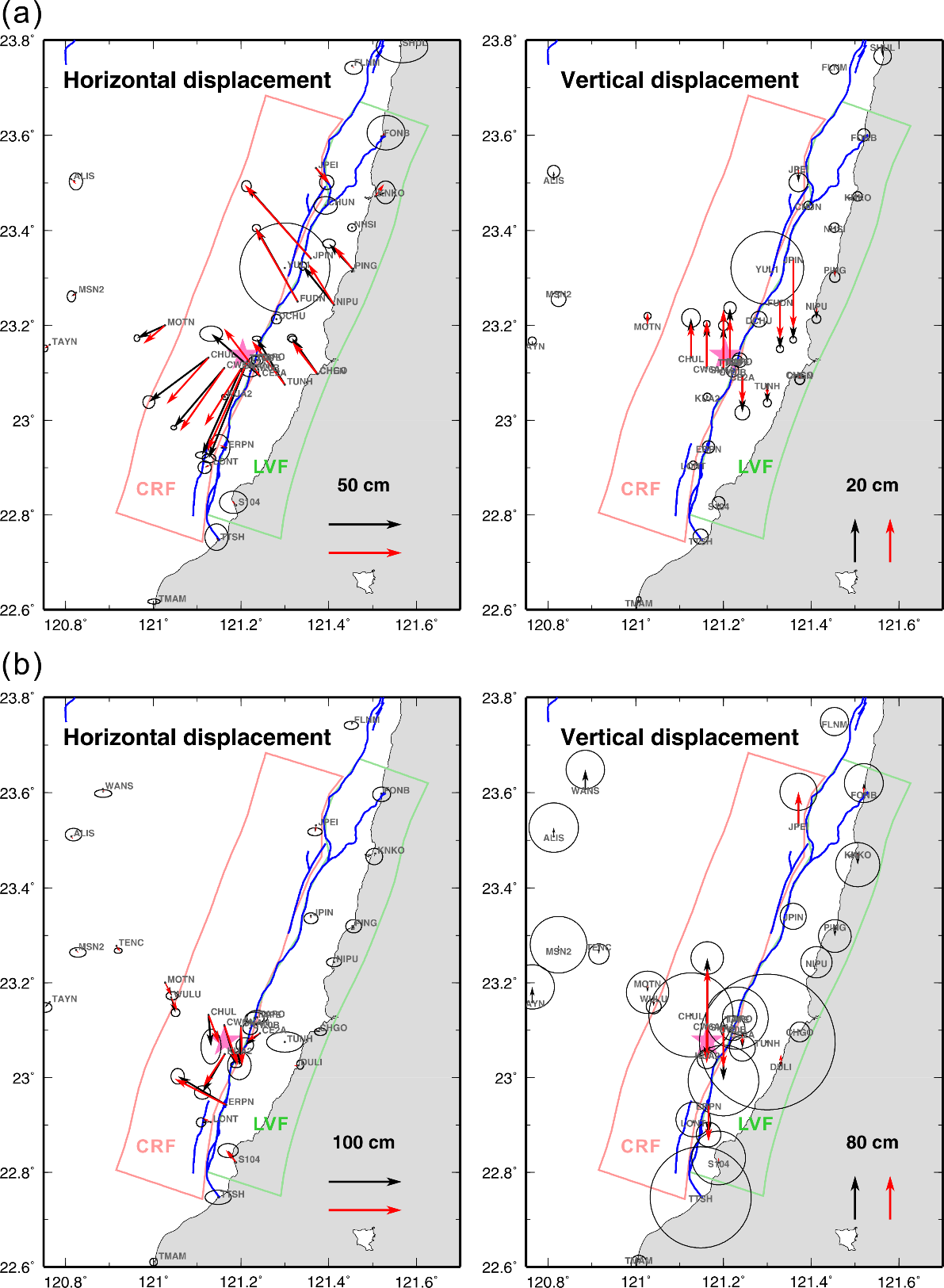


**Figure S3.** Comparison of the observed GNSS coseismic displacements and synthetics: (a) mainshock, and (b) foreshock. Black arrows are observations with ellipses showing associated errors, and red arrows are synthetics. The left panel shows the horizontal component (derived from E-W and N-S components), and the right panel is the vertical component. Active faults in the study region are shown with blue lines. The map was generated by the GMT v.4.3.1 (https://www.generic-mapping-tools.org/).


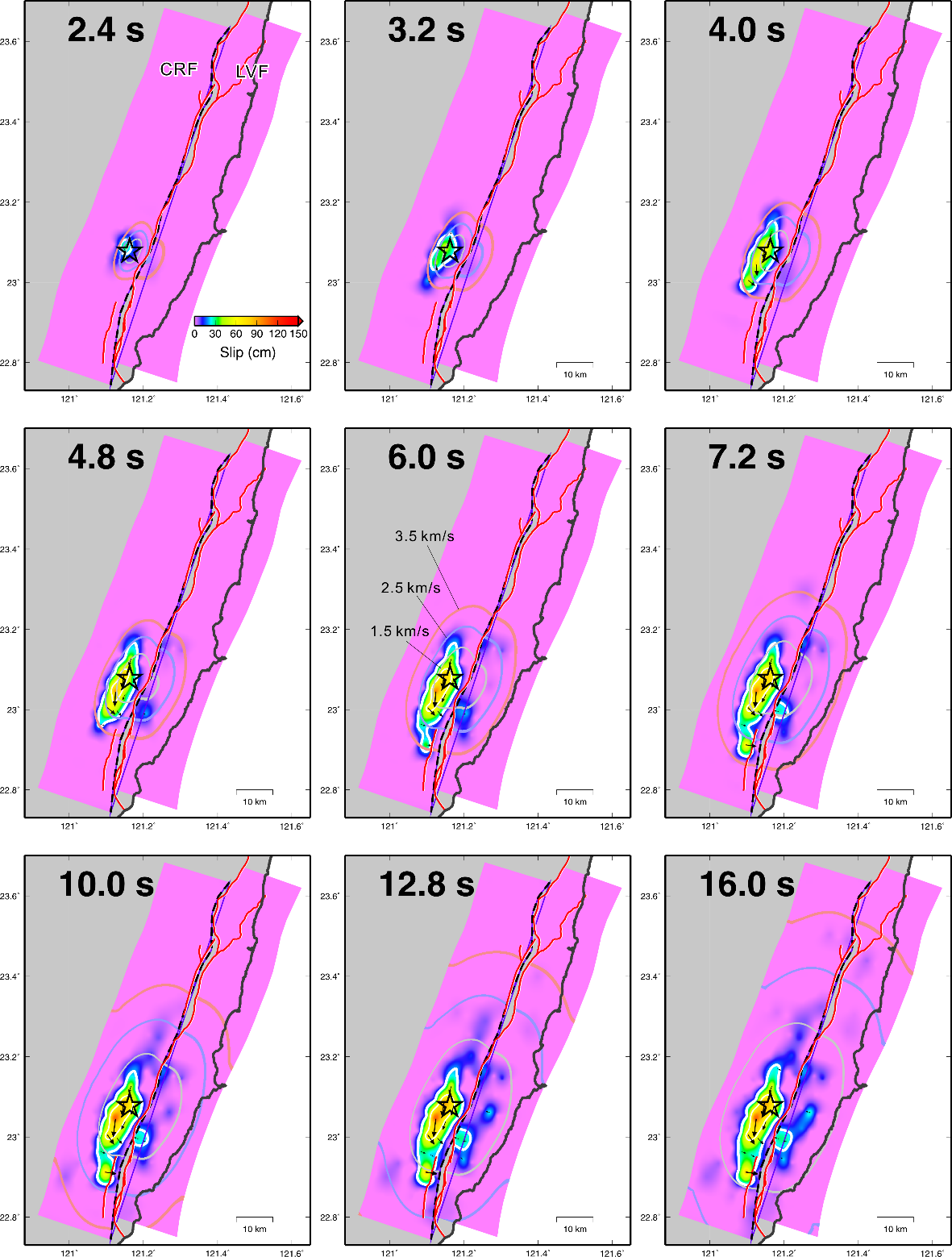


**Figure S4.** Rupture snapshots of the 0917 foreshock. Three reference rupture fronts with constant rupture speed Vr = 3.5, 2.5, and 1.5 km/s are shown with pink, blue and gray contours, respectively. The black open star is the epicenter of the mainshock determined by CWB^37^. The fault plane on the right is the east-dipping Longitudinal Valley Fault (LVF), and on the left is the west-dipping Central Range Fault (CRF). Active faults published by CGS are shown in red lines^36^. White contours show the distribution of accumulated slips. Arrows are the slip vectors on the fault plane. The map was generated by the GMT v.4.3.1 (https://www.generic-mapping-tools.org/).


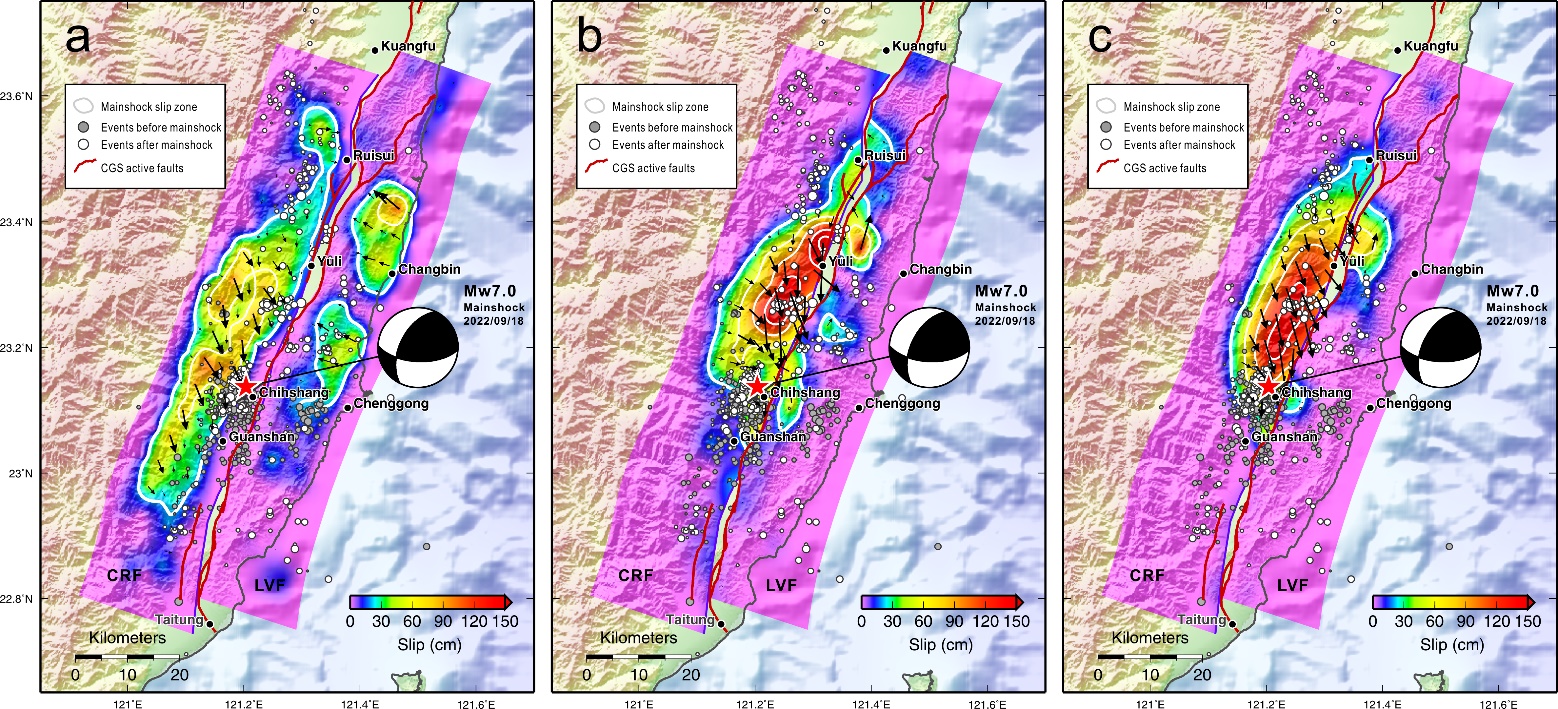


**Figure S5.** The 0918 mainshock slip distributions derived from the inversion with different data sets: (a) Teleseismic body wave, (b) Local ground motion, and (c) GNSS coseismic displacement. The red solid star is the epicenter of the 0918 mainshock determined by CWB^37^. The fault plane on the right is the east-dipping Longitudinal Valley Fault (LVF), and on the left is the west-dipping Central Range Fault (CRF). Active faults published by CGS are shown in red lines^36^. White contours show the slip distributions derived from each data set. Arrows are the slip vectors on the fault plane. Beachballs are the focal mechanisms taken from the RMT CMT report^8^. The events before the mainshock (from 2022/09/17 to 2022/09/18 06:35 that were detected by CWB^37^) are shown in solid gray circles, and the events after the mainshock (from 2022/09/18 06:44 to 2022/09/30 19:51) are presented with white circles. The map was generated by the GMT v.4.3.1 (https://www.generic-mapping-tools.org/).


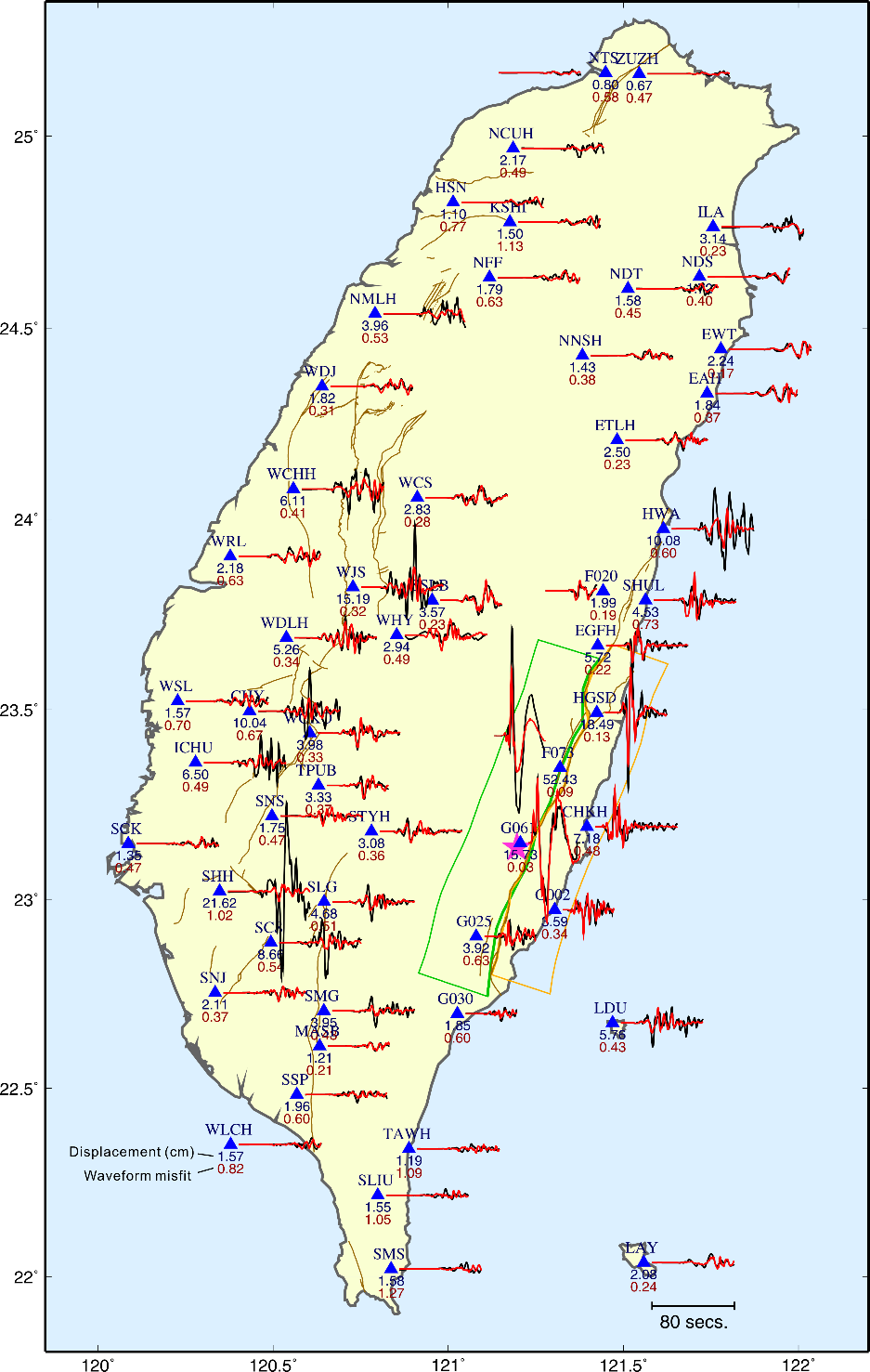


**Figure S6.** Comparison of the island-wide mainshock observed ground motion waveforms and synthetics. Black lines are observations and red lines are synthetics. All the vertical component waveforms are in displacement type, starting from event time and lasting for 80 seconds. A bandpass filtered between 0.05 and 0.5 Hz was employed. The blue and red numbers beneath each local seismic station are the maximum amplitude and waveform misfit, respectively. The misfit of the overall 80 s vertical component displacement waveform is 0.378. The map was generated by the GMT v.4.3.1 (https://www.generic-mapping-tools.org/).

**Table S1.** List of major large events occurred in the 1951 Longitudinal Valley earthquake sequence.

| **LV** | **Date**  (yyyy/mm/dd) | **Time**  (hh:mi) | **Longitude**  (°E) | **Latitude**  (°N) | **Depth**  (km) | **M_L_** | **Strike**  ( ° ) | **Dip**  ( ° ) | **Rake**  ( ° ) |
| --- | --- | --- | --- | --- | --- | --- | --- | --- | --- |
| North  South | 1951/10/22 | 03:29 | 121.725 | 24.075 | 1.0 | 7.0 | 25 | 85 | 73 |
|  | 1951/10/21 | 21:34 | 121.725 | 23.875 | 4.0 | 7.1 | 25 | 85 | 31 |
|  | 1951/10/22 | 05:42 | 121.950 | 23.825 | 18.0 | 6.9 | 291 | 33 | 152 |
|  | 1951/11/24 | 18:50 | 121.350 | 23.275 | 36.0 | 7.0 | 25 | 70 | 40 |
|  | 1951/11/24 | 18:47 | 121.225 | 23.108 | 16.0 | 6.0 | 32 | 70 | 70 |
|  | 1951/11/26 | 06:38 | 121.050 | 22.725 | 49.0 | 5.9 | 8 | 55 | 90 |
|  | 1951/12/05 | 06:58 | 121.375 | 22.725 | 2.0 | 5.8 | 25 | 75 | 40 |

The event time, location, local magnitude, and focal mechanism (strike, dip, and rake) are proposed by Cheng et al.^38-40^.

**Table S2.** The fault plane geometries of CRF and LVF considered in this study. The grid interval is 5 km and the subfault size is 5 km by 5 km.

| **CRF** | | | | |  | **LVF** | | | | |
| --- | --- | --- | --- | --- | --- | --- | --- | --- | --- | --- |
| **Lon.**  (°E) | **Lat.**  (°N) | **Depth**  (km) | **Strike**  (°) | **Dip**  (°) |  | **Lon.**  (°E) | **Lat.**  (°N) | **Depth**  (km) | **Strike**  (°) | **Dip**  (°) |
| 121.102 | 22.770 | 2.30 | 186.22 | 59.86 |  | 121.467 | 23.649 | 1.79 | 27.04 | 59.04 |
| 121.107 | 22.815 | 2.40 | 187.35 | 61.39 |  | 121.439 | 23.612 | 2.11 | 32.53 | 59.23 |
| 121.114 | 22.860 | 2.37 | 188.41 | 61.06 |  | 121.415 | 23.574 | 2.59 | 4.11 | 60.54 |
| 121.122 | 22.905 | 2.16 | 188.24 | 60.84 |  | 121.405 | 23.531 | 3.21 | 1.99 | 60.20 |
| 121.134 | 22.948 | 2.07 | 200.86 | 60.61 |  | 121.408 | 23.487 | 0.98 | 351.41 | 67.54 |
| 121.154 | 22.990 | 2.26 | 207.85 | 60.08 |  | 121.401 | 23.446 | 1.60 | 46.30 | 61.94 |
| 121.176 | 23.030 | 2.45 | 206.86 | 59.98 |  | 121.377 | 23.407 | 2.51 | 36.95 | 60.88 |
| 121.199 | 23.070 | 2.37 | 208.04 | 60.02 |  | 121.359 | 23.365 | 1.85 | 15.48 | 61.34 |
| 121.219 | 23.112 | 2.51 | 198.62 | 60.00 |  | 121.348 | 23.321 | 2.01 | 6.96 | 62.14 |
| 121.232 | 23.155 | 2.29 | 191.24 | 61.03 |  | 121.329 | 23.280 | 2.01 | 32.54 | 63.16 |
| 121.247 | 23.198 | 2.02 | 204.33 | 61.27 |  | 121.303 | 23.242 | 2.27 | 34.30 | 61.08 |
| 121.267 | 23.239 | 2.48 | 206.54 | 59.79 |  | 121.280 | 23.203 | 1.67 | 16.94 | 59.00 |
| 121.286 | 23.281 | 2.47 | 196.80 | 59.95 |  | 121.259 | 23.162 | 2.84 | 26.13 | 61.13 |
| 121.301 | 23.325 | 2.22 | 197.29 | 60.62 |  | 121.240 | 23.120 | 1.73 | 20.27 | 59.65 |
| 121.317 | 23.367 | 2.37 | 203.00 | 59.92 |  | 121.225 | 23.077 | 2.12 | 0.67 | 60.75 |
| 121.336 | 23.409 | 2.21 | 201.77 | 61.34 |  | 121.202 | 23.037 | 2.68 | 39.84 | 60.90 |
| 121.358 | 23.449 | 2.87 | 212.97 | 61.91 |  | 121.186 | 22.995 | 2.99 | 358.00 | 62.65 |
| 121.372 | 23.491 | 2.81 | 179.68 | 61.61 |  | 121.177 | 22.951 | 2.01 | 21.95 | 60.84 |
| 121.373 | 23.536 | 2.22 | 181.21 | 64.75 |  | 121.164 | 22.908 | 2.48 | 18.05 | 60.17 |
| 121.382 | 23.580 | 1.80 | 198.78 | 63.91 |  | 121.153 | 22.863 | 2.04 | 13.70 | 59.55 |
| 121.404 | 23.620 | 2.01 | 216.52 | 59.78 |  | 121.144 | 22.819 | 2.17 | 0.62 | 59.58 |
| 121.075 | 22.778 | 7.26 | 188.75 | 59.96 |  | 121.490 | 23.641 | 6.05 | 26.04 | 59.78 |
| 121.083 | 22.823 | 7.12 | 188.24 | 61.85 |  | 121.463 | 23.604 | 6.54 | 33.20 | 59.33 |
| 121.089 | 22.868 | 7.21 | 187.32 | 61.74 |  | 121.439 | 23.566 | 7.11 | 3.95 | 59.94 |
| 121.097 | 22.912 | 6.99 | 189.55 | 61.87 |  | 121.428 | 23.523 | 7.54 | 2.79 | 60.78 |
| 121.110 | 22.956 | 6.72 | 199.21 | 61.06 |  | 121.431 | 23.479 | 5.61 | 347.05 | 65.55 |
| 121.129 | 22.997 | 6.92 | 207.22 | 60.46 |  | 121.424 | 23.437 | 6.15 | 44.65 | 62.16 |
| 121.151 | 23.038 | 7.07 | 207.36 | 60.17 |  | 121.400 | 23.398 | 7.00 | 36.27 | 61.08 |
| 121.173 | 23.078 | 7.12 | 206.98 | 59.63 |  | 121.382 | 23.356 | 6.29 | 16.05 | 60.30 |
| 121.193 | 23.119 | 7.31 | 200.86 | 59.93 |  | 121.369 | 23.313 | 6.03 | 7.62 | 61.48 |
| 121.207 | 23.162 | 7.10 | 192.61 | 60.77 |  | 121.349 | 23.272 | 6.15 | 33.97 | 59.76 |
| 121.222 | 23.205 | 6.84 | 201.62 | 60.61 |  | 121.324 | 23.234 | 6.32 | 33.12 | 60.79 |
| 121.242 | 23.247 | 7.01 | 206.10 | 59.79 |  | 121.303 | 23.194 | 6.01 | 15.44 | 60.09 |
| 121.260 | 23.288 | 7.24 | 199.13 | 60.01 |  | 121.282 | 23.153 | 7.23 | 25.52 | 60.25 |
| 121.275 | 23.331 | 7.12 | 196.76 | 60.47 |  | 121.263 | 23.112 | 6.15 | 21.13 | 59.31 |
| 121.292 | 23.374 | 6.96 | 201.44 | 60.24 |  | 121.247 | 23.069 | 6.25 | 359.90 | 60.97 |
| 121.311 | 23.416 | 6.98 | 206.02 | 60.31 |  | 121.225 | 23.029 | 7.01 | 41.50 | 61.46 |
| 121.334 | 23.456 | 7.56 | 210.83 | 62.33 |  | 121.209 | 22.988 | 7.39 | 357.00 | 62.06 |
| 121.348 | 23.497 | 7.48 | 183.77 | 62.69 |  | 121.199 | 22.943 | 6.27 | 21.94 | 60.05 |
| 121.351 | 23.542 | 7.06 | 182.71 | 65.38 |  | 121.186 | 22.900 | 6.67 | 18.21 | 60.27 |
| 121.360 | 23.586 | 6.65 | 195.50 | 64.98 |  | 121.175 | 22.856 | 6.16 | 12.40 | 59.95 |
| 121.378 | 23.627 | 6.73 | 208.26 | 61.73 |  | 121.168 | 22.811 | 6.73 | 0.57 | 60.82 |
| 121.049 | 22.787 | 12.07 | 191.26 | 59.55 |  | 121.514 | 23.634 | 10.42 | 27.21 | 60.28 |
| 121.058 | 22.831 | 12.06 | 189.22 | 60.81 |  | 121.486 | 23.596 | 10.84 | 33.99 | 60.89 |
| 121.065 | 22.875 | 11.96 | 187.30 | 62.02 |  | 121.462 | 23.558 | 11.49 | 3.78 | 60.53 |
| 121.073 | 22.920 | 11.77 | 189.92 | 61.28 |  | 121.451 | 23.515 | 11.91 | 3.09 | 60.67 |
| 121.085 | 22.963 | 11.59 | 197.63 | 60.93 |  | 121.454 | 23.470 | 10.02 | 346.51 | 65.74 |
| 121.103 | 23.005 | 11.77 | 206.53 | 59.82 |  | 121.446 | 23.429 | 10.45 | 46.14 | 61.38 |
| 121.125 | 23.046 | 11.86 | 207.64 | 59.86 |  | 121.422 | 23.390 | 11.26 | 34.18 | 61.71 |
| 121.147 | 23.086 | 11.89 | 206.11 | 60.54 |  | 121.404 | 23.348 | 10.50 | 15.63 | 60.82 |
| 121.167 | 23.127 | 12.11 | 202.38 | 60.15 |  | 121.391 | 23.304 | 10.23 | 7.65 | 61.32 |
| 121.182 | 23.170 | 11.85 | 192.83 | 60.71 |  | 121.372 | 23.264 | 10.44 | 33.37 | 60.55 |
| 121.197 | 23.212 | 11.57 | 200.43 | 60.22 |  | 121.347 | 23.226 | 10.67 | 33.91 | 60.55 |
| 121.216 | 23.254 | 11.72 | 205.65 | 60.04 |  | 121.325 | 23.186 | 10.25 | 15.89 | 59.39 |
| 121.234 | 23.296 | 12.03 | 200.59 | 60.15 |  | 121.304 | 23.145 | 11.47 | 24.90 | 60.18 |
| 121.250 | 23.338 | 11.82 | 196.39 | 60.27 |  | 121.285 | 23.104 | 10.31 | 20.67 | 59.71 |
| 121.266 | 23.381 | 11.76 | 201.10 | 60.14 |  | 121.270 | 23.061 | 10.62 | 2.28 | 60.76 |
| 121.285 | 23.422 | 11.71 | 205.61 | 59.95 |  | 121.248 | 23.021 | 11.30 | 40.49 | 61.61 |
| 121.308 | 23.462 | 12.40 | 213.00 | 60.03 |  | 121.231 | 22.980 | 11.60 | 357.88 | 61.77 |
| 121.324 | 23.504 | 12.33 | 187.10 | 62.10 |  | 121.222 | 22.936 | 10.56 | 21.45 | 60.36 |
| 121.330 | 23.548 | 11.80 | 184.55 | 64.84 |  | 121.208 | 22.893 | 10.84 | 17.43 | 60.11 |
| 121.338 | 23.592 | 11.66 | 193.66 | 64.82 |  | 121.197 | 22.849 | 10.36 | 12.47 | 59.80 |
| 121.354 | 23.633 | 11.53 | 202.77 | 62.71 |  | 121.191 | 22.804 | 11.09 | 0.70 | 60.96 |
| 121.023 | 22.795 | 16.81 | 193.43 | 58.73 |  | 121.537 | 23.626 | 14.69 | 27.23 | 60.92 |
| 121.033 | 22.839 | 16.83 | 190.10 | 60.59 |  | 121.510 | 23.588 | 15.27 | 36.35 | 58.03 |
| 121.041 | 22.883 | 16.73 | 187.71 | 61.60 |  | 121.487 | 23.550 | 15.82 | 16.71 | 55.01 |
| 121.049 | 22.928 | 16.43 | 188.86 | 61.45 |  | 121.475 | 23.507 | 16.02 | 3.84 | 55.01 |
| 121.060 | 22.971 | 16.42 | 197.28 | 60.60 |  | 121.477 | 23.462 | 14.37 | 347.67 | 63.31 |
| 121.078 | 23.013 | 16.40 | 204.78 | 60.32 |  | 121.468 | 23.420 | 14.64 | 40.21 | 60.64 |
| 121.099 | 23.053 | 16.60 | 207.44 | 59.72 |  | 121.445 | 23.381 | 15.48 | 32.99 | 56.53 |
| 121.121 | 23.093 | 16.74 | 207.58 | 59.88 |  | 121.427 | 23.339 | 14.89 | 14.54 | 59.65 |
| 121.142 | 23.134 | 16.77 | 202.25 | 59.85 |  | 121.414 | 23.296 | 14.65 | 10.80 | 61.37 |
| 121.157 | 23.177 | 16.62 | 193.43 | 60.90 |  | 121.394 | 23.256 | 14.62 | 33.43 | 60.92 |
| 121.171 | 23.220 | 16.42 | 198.91 | 60.45 |  | 121.370 | 23.217 | 15.17 | 31.35 | 60.67 |
| 121.190 | 23.261 | 16.49 | 206.15 | 60.26 |  | 121.348 | 23.178 | 14.61 | 17.48 | 61.05 |
| 121.209 | 23.303 | 16.69 | 200.64 | 60.39 |  | 121.327 | 23.137 | 15.58 | 26.58 | 56.30 |
| 121.225 | 23.345 | 16.48 | 196.21 | 60.39 |  | 121.308 | 23.096 | 14.75 | 19.99 | 60.99 |
| 121.240 | 23.388 | 16.56 | 200.99 | 60.34 |  | 121.292 | 23.053 | 14.69 | 2.89 | 60.34 |
| 121.259 | 23.429 | 16.43 | 204.36 | 60.27 |  | 121.272 | 23.013 | 15.47 | 41.38 | 57.14 |
| 121.282 | 23.469 | 16.95 | 214.06 | 59.99 |  | 121.255 | 22.972 | 15.70 | 0.25 | 55.81 |
| 121.300 | 23.510 | 17.08 | 191.06 | 61.34 |  | 121.245 | 22.928 | 14.95 | 19.51 | 60.28 |
| 121.307 | 23.554 | 16.85 | 186.37 | 63.59 |  | 121.231 | 22.885 | 15.17 | 19.14 | 59.26 |
| 121.316 | 23.598 | 16.59 | 192.82 | 64.13 |  | 121.220 | 22.842 | 14.75 | 11.86 | 61.56 |
| 121.331 | 23.640 | 16.31 | 199.56 | 63.04 |  | 121.214 | 22.797 | 15.40 | 1.66 | 59.00 |
| 120.996 | 22.803 | 21.58 | 195.79 | 57.61 |  | 121.560 | 23.618 | 19.11 | 29.17 | 60.88 |
| 121.007 | 22.847 | 21.78 | 193.17 | 59.54 |  | 121.535 | 23.580 | 19.41 | 35.13 | 57.97 |
| 121.016 | 22.891 | 21.61 | 186.48 | 60.89 |  | 121.513 | 23.541 | 19.82 | 23.57 | 54.15 |
| 121.024 | 22.935 | 21.31 | 189.69 | 61.11 |  | 121.502 | 23.498 | 19.87 | 1.05 | 53.90 |
| 121.035 | 22.979 | 21.19 | 195.58 | 60.52 |  | 121.500 | 23.454 | 18.77 | 358.72 | 62.31 |
| 121.052 | 23.021 | 21.25 | 205.30 | 60.18 |  | 121.489 | 23.412 | 18.84 | 27.24 | 61.91 |
| 121.073 | 23.061 | 21.35 | 206.08 | 59.99 |  | 121.469 | 23.372 | 19.51 | 26.51 | 57.61 |
| 121.095 | 23.101 | 21.47 | 208.26 | 59.63 |  | 121.451 | 23.330 | 19.32 | 16.92 | 58.84 |
| 121.116 | 23.142 | 21.55 | 202.71 | 59.56 |  | 121.436 | 23.288 | 19.06 | 17.33 | 61.33 |
| 121.132 | 23.184 | 21.40 | 194.79 | 60.39 |  | 121.417 | 23.247 | 19.11 | 30.49 | 61.56 |
| 121.146 | 23.227 | 21.13 | 197.05 | 60.35 |  | 121.393 | 23.209 | 19.49 | 29.47 | 58.54 |
| 121.164 | 23.268 | 21.26 | 207.06 | 59.97 |  | 121.371 | 23.169 | 19.17 | 23.51 | 60.25 |
| 121.183 | 23.310 | 21.54 | 200.34 | 59.93 |  | 121.350 | 23.129 | 19.46 | 26.95 | 58.50 |
| 121.199 | 23.352 | 21.35 | 196.60 | 60.58 |  | 121.332 | 23.088 | 19.39 | 19.62 | 59.49 |
| 121.215 | 23.394 | 21.22 | 200.84 | 60.58 |  | 121.315 | 23.046 | 19.12 | 17.92 | 60.45 |
| 121.233 | 23.436 | 21.22 | 203.47 | 60.50 |  | 121.296 | 23.005 | 19.43 | 30.21 | 57.67 |
| 121.256 | 23.476 | 21.49 | 214.60 | 59.71 |  | 121.280 | 22.964 | 19.64 | 10.95 | 56.44 |
| 121.274 | 23.517 | 22.04 | 193.68 | 59.79 |  | 121.268 | 22.920 | 19.31 | 16.08 | 59.75 |
| 121.284 | 23.560 | 21.63 | 188.27 | 61.96 |  | 121.255 | 22.878 | 19.53 | 16.00 | 59.13 |
| 121.294 | 23.604 | 21.36 | 192.85 | 63.14 |  | 121.244 | 22.834 | 19.44 | 11.22 | 60.21 |
| 121.308 | 23.646 | 21.13 | 197.79 | 62.99 |  | 121.237 | 22.790 | 19.42 | 1.43 | 60.38 |
| 120.968 | 22.812 | 26.29 | 197.91 | 56.09 |  | 121.583 | 23.611 | 23.51 | 27.95 | 61.02 |
| 120.981 | 22.855 | 26.43 | 194.84 | 56.92 |  | 121.560 | 23.572 | 23.66 | 29.86 | 58.36 |
| 120.991 | 22.899 | 26.26 | 187.78 | 58.76 |  | 121.540 | 23.532 | 23.90 | 20.88 | 55.27 |
| 120.999 | 22.943 | 26.11 | 189.94 | 60.35 |  | 121.528 | 23.489 | 23.69 | 5.00 | 56.67 |
| 121.011 | 22.986 | 25.81 | 195.51 | 61.23 |  | 121.522 | 23.446 | 23.17 | 7.08 | 61.95 |
| 121.027 | 23.028 | 25.96 | 204.40 | 60.79 |  | 121.510 | 23.404 | 23.22 | 22.18 | 62.14 |
| 121.048 | 23.069 | 25.99 | 206.17 | 60.13 |  | 121.492 | 23.363 | 23.61 | 24.07 | 59.37 |
| 121.069 | 23.109 | 26.17 | 207.48 | 59.65 |  | 121.474 | 23.321 | 23.53 | 19.12 | 59.54 |
| 121.090 | 23.149 | 26.23 | 203.28 | 58.99 |  | 121.458 | 23.280 | 23.48 | 20.29 | 61.30 |
| 121.106 | 23.191 | 26.25 | 195.89 | 59.79 |  | 121.438 | 23.239 | 23.38 | 27.63 | 61.44 |
| 121.121 | 23.234 | 25.87 | 196.80 | 61.37 |  | 121.416 | 23.200 | 23.64 | 28.50 | 59.47 |
| 121.139 | 23.276 | 25.86 | 206.28 | 60.91 |  | 121.394 | 23.161 | 23.55 | 25.50 | 59.64 |
| 121.157 | 23.317 | 26.29 | 201.34 | 59.77 |  | 121.374 | 23.120 | 23.75 | 25.15 | 59.05 |
| 121.174 | 23.359 | 26.07 | 197.62 | 60.77 |  | 121.355 | 23.079 | 23.65 | 20.21 | 59.06 |
| 121.190 | 23.401 | 25.95 | 199.84 | 61.16 |  | 121.338 | 23.038 | 23.52 | 20.21 | 59.83 |
| 121.208 | 23.443 | 25.90 | 203.26 | 60.86 |  | 121.320 | 22.997 | 23.60 | 23.53 | 58.34 |
| 121.229 | 23.483 | 26.13 | 211.25 | 59.23 |  | 121.304 | 22.955 | 23.63 | 15.59 | 57.73 |
| 121.248 | 23.524 | 26.59 | 196.94 | 57.08 |  | 121.291 | 22.913 | 23.58 | 16.45 | 59.35 |
| 121.260 | 23.567 | 26.30 | 190.87 | 60.40 |  | 121.278 | 22.870 | 23.71 | 15.55 | 59.28 |
| 121.271 | 23.610 | 26.13 | 193.70 | 62.24 |  | 121.267 | 22.827 | 23.72 | 11.09 | 59.59 |
| 121.285 | 23.653 | 25.92 | 197.15 | 62.70 |  | 121.260 | 22.783 | 23.68 | 4.64 | 60.66 |
| 120.938 | 22.821 | 31.03 | 199.35 | 54.72 |  | 121.605 | 23.604 | 27.78 | 26.11 | 61.03 |
| 120.953 | 22.864 | 31.00 | 195.53 | 55.54 |  | 121.584 | 23.564 | 27.89 | 26.16 | 59.00 |
| 120.964 | 22.907 | 30.95 | 190.75 | 57.45 |  | 121.566 | 23.523 | 28.06 | 19.04 | 57.27 |
| 120.974 | 22.951 | 30.79 | 191.71 | 59.59 |  | 121.554 | 23.481 | 27.99 | 9.81 | 58.72 |
| 120.986 | 22.994 | 30.70 | 196.51 | 60.92 |  | 121.544 | 23.438 | 27.61 | 11.50 | 61.79 |
| 121.002 | 23.036 | 30.77 | 202.84 | 60.93 |  | 121.531 | 23.395 | 27.62 | 19.92 | 62.08 |
| 121.022 | 23.077 | 30.81 | 205.65 | 60.20 |  | 121.514 | 23.354 | 27.79 | 22.20 | 60.59 |
| 121.043 | 23.117 | 30.88 | 206.33 | 59.51 |  | 121.497 | 23.313 | 27.87 | 20.60 | 60.52 |
| 121.063 | 23.157 | 31.00 | 203.43 | 58.98 |  | 121.479 | 23.272 | 27.72 | 21.88 | 61.39 |
| 121.081 | 23.199 | 30.85 | 198.10 | 59.71 |  | 121.460 | 23.231 | 27.80 | 25.87 | 61.29 |
| 121.096 | 23.241 | 30.76 | 198.28 | 61.36 |  | 121.439 | 23.192 | 27.93 | 27.14 | 60.09 |
| 121.113 | 23.283 | 30.81 | 203.39 | 61.36 |  | 121.417 | 23.152 | 27.85 | 25.95 | 59.57 |
| 121.132 | 23.324 | 30.88 | 201.90 | 60.34 |  | 121.397 | 23.112 | 27.96 | 24.26 | 58.98 |
| 121.149 | 23.366 | 30.81 | 198.84 | 60.84 |  | 121.378 | 23.071 | 27.85 | 20.90 | 58.97 |
| 121.165 | 23.408 | 30.77 | 199.61 | 61.34 |  | 121.361 | 23.030 | 27.82 | 20.70 | 59.39 |
| 121.182 | 23.449 | 30.81 | 202.33 | 61.12 |  | 121.344 | 22.989 | 27.87 | 20.75 | 58.76 |
| 121.202 | 23.490 | 30.84 | 206.22 | 59.55 |  | 121.329 | 22.947 | 27.97 | 17.38 | 58.54 |
| 121.221 | 23.531 | 31.02 | 199.97 | 57.66 |  | 121.314 | 22.905 | 27.80 | 16.97 | 59.15 |
| 121.235 | 23.573 | 30.92 | 194.14 | 59.69 |  | 121.301 | 22.862 | 27.90 | 15.31 | 59.10 |
| 121.247 | 23.616 | 30.92 | 195.15 | 61.63 |  | 121.290 | 22.819 | 27.92 | 11.24 | 59.30 |
| 121.261 | 23.659 | 30.84 | 197.25 | 62.38 |  | 121.282 | 22.775 | 27.82 | 6.88 | 60.44 |

**Table S3.** GSN stations considered in this study and their signal-to-noise ratio (SNR). The SNR for each vertical component waveform was determined by using 10 seconds of processed data before the first P arrival as noise and 35 seconds of processed data after the P arrival as the signal. The signal and noise are first averaged and squared. Then the signal is divided by the noise to obtain a quantity proportional to power. Finally, the common logarithm of this ratio is taken as SNR. Only data with SNR greater than 0.75 (approximately 5 times larger than noise) was considered to use in the inversion. The stations used in the source inversions of mainshock and foreshock are shown with gray color background in the table.

| **Station** | **Net** | **Loc.** | **Lon.**  (°E) | **Lat.**  (°N) | **Distance**  (degree) | **Azimuth**  ( ° ) | **0918 SNR** (dB) | **0917 SNR** (dB) |
| --- | --- | --- | --- | --- | --- | --- | --- | --- |
| AAK | II | 00 | 74.494 | 42.638 | 43.3 | 308.2 | 3.53 | 2.91 |
| ABPO | II | 00 | 47.229 | -19.018 | 83.8 | 246.3 | 2.95 | 2.48 |
| ADK | IU | 00 | -176.684 | 51.882 | 55.0 | 42.0 | 3.09 | 1.66 |
| AFI | IU | 00 | -171.783 | -13.909 | 75.3 | 112.5 | 2.27 | 1.77 |
| ALE | II | 00 | -62.350 | 82.503 | 74.6 | 0.5 | 3.07 | 3.45 |
| ANTO | IU | 00 | 32.793 | 39.868 | 74.6 | 307.0 | 3.91 | 2.22 |
| ARTI | II | 00 | 58.385 | 56.388 | 56.2 | 323.4 | 3.79 | 3.46 |
| BFO | II | 00 | 8.330 | 48.330 | 87.2 | 322.1 | 2.23 | 2.34 |
| BILL | IU | 00 | 166.453 | 68.065 | 52.7 | 19.6 | 3.27 | 2.55 |
| BORG | II | 00 | -21.327 | 64.747 | 87.8 | 344.9 | 1.95 | 1.00 |
| CASY | IU | 00 | 110.535 | -66.279 | 89.7 | 184.4 | 1.49 | 0.88 |
| COCO | II | 00 | 96.835 | -12.190 | 42.7 | 216.9 | 2.44 | 1.45 |
| COLA | IU | 00 | -147.862 | 64.874 | 69.7 | 27.1 | 3.55 | 2.72 |
| CTAO | IU | 00 | 146.250 | -20.088 | 49.5 | 148.6 | 3.14 | 2.33 |
| ESK | II | 00 | -3.205 | 55.317 | 88.8 | 331.9 | 1.76 | 1.43 |
| FUNA | IU | 00 | 179.197 | -8.526 | 64.9 | 112.2 | 0.42 | 0.56 |
| GNI | IU | 00 | 44.741 | 40.148 | 65.6 | 305.0 | 3.58 | 2.94 |
| GRFO | IU | 00 | 11.220 | 49.691 | 84.8 | 322.3 | 2.53 | 2.26 |
| HNR | IU | 00 | 159.947 | -9.439 | 49.9 | 126.3 | 3.40 | 3.27 |
| JOHN | IU | 00 | -169.529 | 16.733 | 64.9 | 81.8 | 1.77 | 0.79 |
| KBS | IU | 00 | 11.938 | 78.915 | 71.1 | 348.9 | 3.76 | 3.19 |
| KDAK | II | 00 | -152.583 | 57.783 | 68.7 | 35.0 | 2.99 | 1.98 |
| KEV | IU | 00 | 27.003 | 69.757 | 70.0 | 338.3 | 2.98 | 2.42 |
| KIP | IU | 00 | -158.011 | 21.420 | 73.8 | 73.4 | 2.11 | 0.87 |
| KIV | II | 00 | 42.686 | 43.955 | 66.5 | 309.4 | 3.63 | 3.40 |
| KMBO | IU | 00 | 37.252 | -1.127 | 85.2 | 266.7 | 2.63 | 2.68 |
| KONO | IU | 00 | 9.598 | 59.649 | 80.7 | 331.4 | 2.16 | 2.14 |
| KURK | II | 00 | 78.620 | 50.715 | 43.1 | 320.7 | 3.30 | 3.19 |
| KWJN | II | 00 | 167.537 | 9.287 | 46.3 | 99.5 | 2.69 | 2.17 |
| LVZ | II | 00 | 34.651 | 67.898 | 67.6 | 335.9 | 2.92 | 2.47 |
| MA2 | IU | 00 | 150.770 | 59.576 | 41.9 | 22.0 | 4.06 | 3.86 |
| MAKZ | IU | 00 | 81.977 | 46.808 | 39.5 | 316.6 | 2.82 | 3.34 |
| MBWA | IU | 00 | 119.731 | -21.159 | 44.2 | 182.2 | 2.88 | 2.83 |
| MCQ | AU | 00 | 158.940 | -54.498 | 84.0 | 159.0 | 0.12 | 0.35 |
| MSEY | II | 00 | 55.479 | -4.674 | 70.1 | 255.6 | 2.77 | 2.56 |
| MSVF | II | 00 | 178.053 | -17.745 | 68.9 | 121.3 | 2.75 | 1.32 |
| NWAO | IU | 00 | 117.239 | -32.928 | 56.1 | 184.2 | 2.60 | 2.13 |
| OBN | II | 00 | 36.567 | 55.115 | 68.5 | 322.0 | 3.28 | 2.09 |
| PALK | II | 00 | 80.702 | 7.273 | 42.2 | 254.6 | 2.82 | 3.16 |
| PET | IU | 00 | 158.650 | 53.023 | 41.1 | 33.9 | 3.40 | 2.65 |
| PMG | IU | 00 | 147.160 | -9.405 | 41.2 | 139.2 | 3.36 | 3.26 |
| POHA | IU | 00 | -155.533 | 19.757 | 76.6 | 74.2 | 2.01 | 1.03 |
| RAR | IU | 00 | -159.773 | -21.212 | 88.8 | 113.7 | 1.63 | 0.27 |
| RAYN | II | 00 | 45.503 | 23.523 | 69.0 | 287.3 | 2.99 | 3.33 |
| SFJD | IU | 00 | -50.621 | 66.996 | 90.0 | 356.9 | 1.74 | 0.79 |
| SIMI | II | 00 | 69.008 | 38.658 | 47.0 | 301.9 | 3.39 | 3.24 |
| SNZO | IU | 00 | 174.704 | -41.309 | 81.2 | 142.3 | 1.92 | 1.12 |
| TARA | IU | 00 | 172.923 | 1.355 | 54.6 | 105.8 | 2.64 | 2.84 |
| TAU | II | 00 | 147.320 | -42.910 | 70.1 | 160.0 | 1.36 | 1.48 |
| TIXI | IU | 00 | 128.867 | 71.634 | 48.7 | 3.2 | -0.47 | -0.05 |
| TLY | II | 00 | 103.644 | 51.681 | 31.6 | 338.8 | 1.18 | 1.82 |
| UOSS | II | 00 | 56.204 | 24.945 | 59.1 | 286.1 | 3.13 | 2.91 |
| WAKE | IU | 00 | 166.652 | 19.283 | 42.4 | 86.4 | 1.66 | 0.98 |
| WRAB | II | 00 | 134.360 | -19.934 | 44.8 | 162.5 | 2.95 | 3.03 |
| XMAS | IU | 00 | -157.446 | 2.045 | 81.3 | 91.6 | 1.88 | 1.74 |
| YAK | IU | 00 | 129.680 | 62.031 | 39.3 | 6.2 | 3.36 | 2.82 |
| YSS | IU | 00 | 142.760 | 46.959 | 29.4 | 30.7 | 2.12 | 3.42 |

**Table S4.** Misfits with varied maximum rupture velocity (V_rmax_) of the mainshock.

| **Data set** | **V_rmax_ 5.0**  km/s | **V_rmax_ 4.0**  km/s | **V_rmax_ 3.0**  km/s | **V_rmax_ 2.8**  km/s | **V_rmax_ 2.0**  km/s | **V_rmax_ 1.0**  km/s |
| --- | --- | --- | --- | --- | --- | --- |
| Teleseismic | 0.222 | 0.203 | 0.187 | 0.187 | 0.199 | 0.362 |
| Local | 0.071 | 0.056 | 0.052 | 0.051 | 0.061 | 0.275 |
| GNSS | 0.035 | 0.035 | 0.034 | 0.034 | 0.034 | 0.052 |
| TOTAL | 0.109 | 0.098 | 0.091 | 0.090 | 0.098 | 0.230 |

**Video S1.** Rupture snapshots of the 0918 mainshock. Three reference rupture fronts with constant rupture speed Vr = 3.5, 2.5, and 1.5 km/s are shown with pink, blue and gray contours, respectively. The black open star is the epicenter of the mainshock determined by CWB^37^. The fault plane on the right is the east-dipping Longitudinal Valley Fault (LVF), and on the left is the west-dipping Central Range Fault (CRF). Active faults published by CGS are shown in red lines^36^. White contours show the distribution of accumulated slips. Arrows are the slip vectors on the fault plane.

**Supplementary Methods**

**The spatial and temporal resolution of the inversion**

Based on the approach of multiple time windows proposed by Hartzell and Heaton^32^, the spatial and temporal resolution in the inversion is highly interrelated. Here we use an example shown in Supplementary Fig. S7 to discuss. We assume a displacement waveform with a period between 2-100s (Fig. S7a). The grid space of the two subfaults is set to 5 km (Fig. S7b), and 24 time windows are set with an interval of 0.4 s. Each subfault can slip at the first and/or later time windows when the rupture front passes through. The rupture velocity is set to 2.5 km/s, and thus it takes 2.0 seconds for the rupture to propagate from subfault 1 to subfault 2.


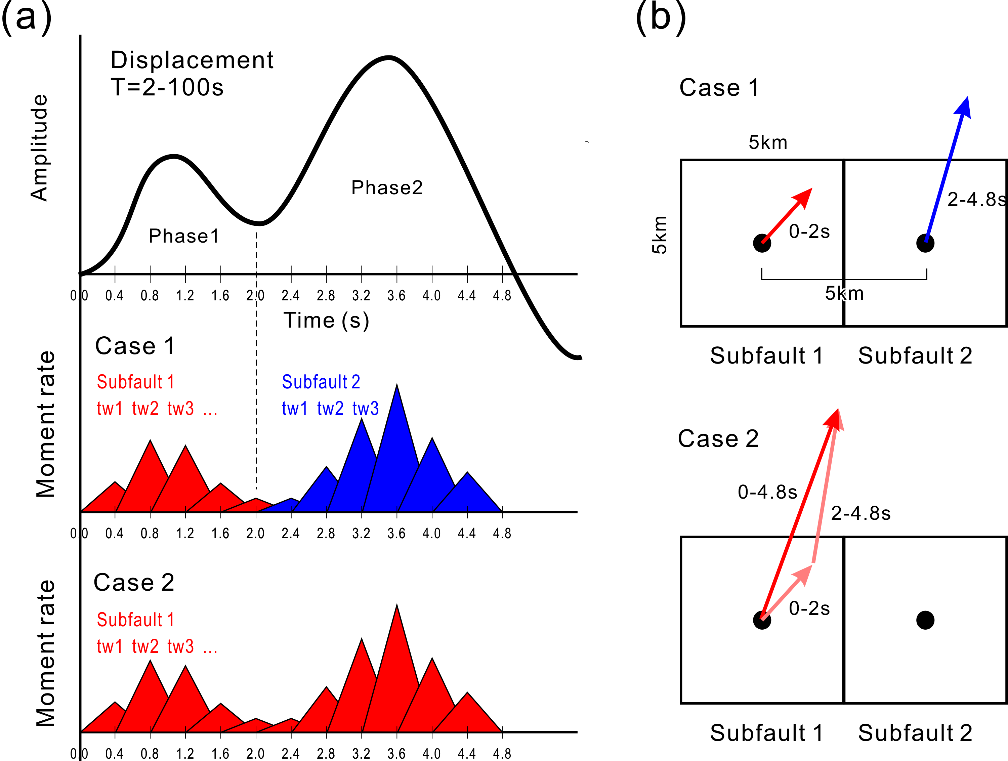


**Figure S7.** The multiple time windows approach. (a) The upper panel is the assumed displacement waveform with a period between 2-100s. The lower panels show two cases of moment rate functions based on multiple time windows (tw). (b) The subfaults and their slips in the two cases.

Two different scenarios can explain the waveform. In the first case, the slip occurs on subfault 1 between 0-2 s first. The rupture takes 2 seconds to propagate from subfault 1 to subfault 2 and then causes the slip to happen on subfault 2 during 2 and 4.8 seconds. In the second case, the slip occurs on subfault 1 first, but the rupture does not propagate outward. Instead, the seismic energy releases on the later time windows at subfault 1 in every 0.4 s that accumulates slip until the 4.8 seconds. It is noted that phase 1 in the waveform lasts approximately 0-2 seconds; its shape can be described by a combination of five 0.8s triangular moment rate functions, each overlapping 0.4 seconds. Therefore, in these two cases, even though the smallest period of the waveform is 2 s, the temporal resolution can still be shorter than 2 s depending on the interval of the time window. Furthermore, the spatial resolution is not constrained by the high bound of the filter, i.e. the shortest period (2 s) multiplied by rupture velocity (2.5 km/s), because the slip can continue occurring at the same subfault (Case 2) or propagated to the vicinity subfault (Case 1). In practical situations, the inversion with multiple time windows approach can help identify which scenario is better if the data sets have good azimuth coverage and broad frequency content, such as the joint inversion with teleseismic data, local strong motion data, and GNSS coseismic displacement.

According to this discussion, the spatial resolution in our study is 5 km which is based on the grid interval (Supplementary Table S2). The temporal resolution is 0.4 s which is according to the interval of multiple time windows. The details of how slips accumulated on subfaults and propagated forward during the mainshock can be found in Supplementary Fig. S8 (a full rupture process animation can be seen in Supplementary Video S1). The inversion parameters used in this study and other published papers are also provided in Supplementary Table S5 for reference.


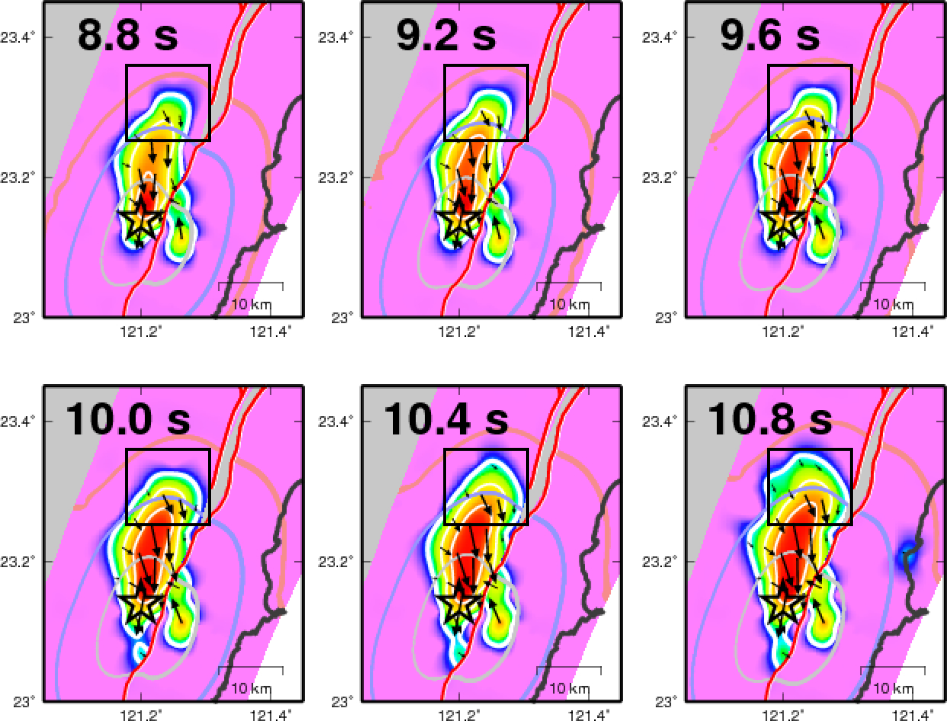


**Figure S8.** Rupture snapshots of the mainshock. Arrows are the slip on the fault plane. Black rectangular indicates how the slip accumulated on the subfault and propagated forward every 0.4 seconds.

**Table S5.** Parameters of this and other source inversion studies

| **Event & Authors** | **Magnitude** | **Subfault size** (km^2^) | **Frequency** (Teleseismic) | **Resampled**  (Teleseismic) | **Data length**  (Teleseismic) | **Time window interval** |
| --- | --- | --- | --- | --- | --- | --- |
| 2022 earthquake sequence  This study | Mw 7.0  Mw 6.6 | 5 x 5 | 0.01 - 0.5 Hz | 0.2 s | 45 s  40 s | 0.4 s |
| 1979 Imperial Valley earthquake  Hartzell and Heaton^32^ | ML 6.6 | 3 x 2.5 -  3 x 3 | 0.016 - 1.0 Hz | 0.25 s | 30-35 s | 0.5 s |
| 2019 Ridgecrest earthquake  Chen et al.^41^ | Mw 7.1 | 2.5 x 2.5 | 0.005 - 0.4 Hz | -- | 120 s | 1.5 s |
| 2019 Albania Earthquake  Papadopoulos et al.^42^ | Mw 6.4 | 1.6 x 2.0 | 0.04 - 0.5 Hz | 0.2 s | 30 s | 0.5 s |
| 2020 Puerto Rico earthquake  Liu et al.^43^ | Mw 6.4 | 1.2 x 1.2 | 0.003 - 1.0 Hz | -- | 35 s | -- |
| 2020 Shumagin earthquake  Ye et al.^44^ | Mw 7.8 | 10 x 10 | 0.005 - 0.9 Hz | -- | 100 s | 2.0 s |
| 2021 Maduo earthquake  Yue et al.^45^ | Mw 7.4 | 3.9 x 2.9 | 0.01 - 0.95 Hz | 0.5 s | 100 s | 2.0 s |

**References**

1. Cheng, S. N., Yeh, Y. T. & Yu, M. S. The 1951 Taitung earthquake in Taiwan. *J. Geol. Soc. China* **39**, 267-285 (1996).
2. Cheng, S. N., Yu, T. T., Yeh, Y. T. & Chang, Z. S. Relocation of the 1951 Hualien, Taitung earthquake sequence (in Chinese). *Conference on Weather Analysis and Forecasting, Proceedings of Marine Meteorology and Seismology, in Commemoration of 100 Years of Weather Observation in the Taiwan Area*, pp. 690-699 (1997).
3. Cheng, S. N., Wang, T. B., Lin, T. W., Chiang, C. H., Establishment of earthquake catalog in Taiwan region (II) (in Chinese). *A Compilation of Seismic Technical Reports of the Central Weather Bureau*, **57**, 483-501 (2011).
4. Chen, K., Avouac, J. P., Aati, S., Milliner, C., Zheng, F. & Shi, C. Cascading and pulse-like ruptures during the 2019 Ridgecrest earthquakes in the Eastern California Shear Zone. *Nature communications* **11**, 22 (2020).
5. Papadopoulos, G. A., Agalos, A., Carydis, P., Lekkas, E., Mavroulis, S. & Triantafyllou, I. The 26 November 2019 Mw 6.4 Albania Destructive Earthquake. *Seismol. Res. Lett.* **91**, 3129–3138 (2020).
6. Liu, C., Lay, T., Wang, Z., & Xiong, X. Rupture process of the 7 January 2020, MW 6.4 Puerto Rico earthquake. *Geophysical Research Letters* **47**, e2020GL087718 (2020).
7. Ye, L., Lay, T., Kanamori, H., Yamazaki, Y. & Cheung, K. F. The 22 July 2020 Mw 7.8 Shumagin seismic gap earthquake: Partial rupture of a weakly coupled megathrust. *Earth and Planetary Science Letters* **562**, 116879 (2021).
8. Yue, H. et al. Rupture process of the 2021 M7. 4 Maduo earthquake and implication for deformation mode of the Songpan-Ganzi terrane in Tibetan Plateau. *Proceedings of the National Academy of Sciences* **119**, e2116445119 (2022).
